# Polysaccharides from *Elsholtzia rugulosa*: structural characteristics and treating functional dyspepsia via gut microbiome in conjunction with targeting the PI3K/Akt/cGMP-PKG pathways

**DOI:** 10.3389/fnut.2026.1833860

**Published:** 2026-08-03

**Authors:** Run Ai, Xue-Ying Xu, Afsar Khan, Jia-Mei Geng, Wen-Hong Tan, Yi-Fen Wang, Lu Liu

**Affiliations:** 1 Yunnan Yunzhong Institute of Nutrition and Health, College of Chinese Medicine, Yunnan University of Chinese Medicine, Kunming, Yunnan, China; 2 Department of Chemistry, COMSATS University Islamabad, Abbottabad Campus, Abbottabad, Pakistan

**Keywords:** *Elsholtzia rugulosa*, functional dyspepsia, gut microbiome, PI3K/Akt/cGMP-PKG pathways, polysaccharides

## Abstract

**Background:**

Functional dyspepsia (FD) is a common gastrointestinal disorder characterized by symptoms such as epigastric pain, bloating, and nausea. Current treatments have limited efficacy and significant side effects. *Elsholtzia rugulosa*, a traditional medicinal plant, is used to treat gastrointestinal disorders, and its polysaccharides (ERP) may offer therapeutic potential for FD.

**Objective:**

This study aimed to characterize the structural properties of *Elsholtzia rugulosa* polysaccharides (ERP) and explore their mechanism in treating FD.

**Materials and methods:**

Neutral ERP-N and acidic ERP-A were isolated from *Elsholtzia rugulosa*. Their structural features were analyzed, and FD rats were treated with ERP. Gastric pathology, gut microbiota, and the PI3K/Akt/cGMP-PKG pathway were evaluated. RAW264.7 cells were used to assess ERP’s immunomodulatory effects.

**Results:**

Structural Analysis: ERP-N (13,415 Da) and ERP-A (3,691 Da) exhibited distinct monosaccharide compositions and linkages. Therapeutic Effects: ERP improved FD symptoms, reduced inflammation, regulated gut microbiota, and activated the PI3K/Akt/cGMP-PKG pathway in rats. Immunomodulatory Activity: ERP-N promoted the secretion of nitric oxide (NO), TNF-*α*, and IL-6 in RAW264.7 cells, demonstrating immunomodulatory effects.

**Conclusion:**

ERP may alleviate FD in association with gut microbiota modulation and changes in the PI3K/Akt/cGMP-PKG-related pathway. ERP-N also exhibits immunomodulatory activity, supporting the traditional use of *Elsholtzia rugulosa* in gastrointestinal health and offering potential for FD therapy development.

## 1 Introduction

Functional dyspepsia (FD), named as “Shi Ji” in traditional Chinese medicine, is a group of clinical syndromes of non-organic diseases, specifically manifested as epigastric pain, upper abdominal bloating, early satiety, belching, loss of appetite, nausea, vomiting, and other discomfort symptoms (1, 2). The treatment cycle of FD is long, and the side effects of existing drugs are high (3). At the same time, there is a trend of increasing incidence rate in younger patients (4). Due to its chronic medical expenditure, it has seriously affected people’s normal work and life (5). Modern pharmacological studies have shown that the pathophysiology and progression of FD are related to impaired gastric regulatory function (6, 7) and intestinal microbiota disturbance (8). Therefore, improving gastrointestinal motility disorders (9) and regulating intestinal flora (10) have become effective strategies for treating functional dyspepsia.

*Elsholtzia rugulosa* Hemsl. is a renowned medicinal and edible plant predominantly found in southwestern China (11). In traditional medicine, it aligns with the stomach meridian and finds application in the treatment of gastrointestinal ailments, including acute gastroenteritis and indigestion (12). Locally, it is frequently utilized for the preparation of herbal teas aimed at treating gastrointestinal disorders and indigestion (13). Furthermore, numerous studies have emphasized the pivotal role of natural polysaccharides in gastrointestinal health. A water-soluble polysaccharide, isolated from Chinese yams, has been found to modulate the composition of gut microbiota (14). Polysaccharides have been shown to ameliorate gastric motility dysfunction through the enhancement of gastric satiety (15). The above data suggest that polysaccharide derived from *E. rugulosa* (ERP) may be a good source for the treatment of FD, and the results of the current study also confirm this hypothesis.

The PI3K/Akt and cGMP-PKG-related signaling pathways are closely associated with gastrointestinal physiological regulation, including inflammatory responses, mucosal barrier function, smooth muscle activity, and gastrointestinal motility. These processes are also influenced by gut microbiota through microbial metabolites, immune modulation, and inflammatory mediators. Therefore, alterations in gut microbiota may be associated with host signaling responses involved in FD-related gastric dysfunction. Based on this rationale, the present study integrated 16S rRNA sequencing, transcriptomic analysis, and pathway-related protein detection to explore whether ERP-induced improvements in FD are accompanied by gut microbiota remodeling and changes in PI3K/Akt/cGMP-PKG-related signaling.

In this study, we first evaluated the structural characterization of two homogeneous polysaccharides, a neutral polysaccharide (ERP-N) and an acidic polysaccharide (ERP-A), purified from ERP. Subsequently, we used transcriptome sequencing combined with 16S rRNA gene sequencing experiments to assess the roles of gastric motility function and gut microbiota in ERP-treated rats having FD. The experimental results showed that ERP significantly improved the impaired gastric regulatory function and gut microbiota abundance caused by FD, enhancing the abundance of beneficial gut microbiota such as Firmicutes, Proteobacteria, and Patescibacteria by activating the PI3K/Akt/cGMP-PKG signaling pathway. Additionally, *in vitro* studies suggested that ERP-N possesses potential immunoregulatory activity. Taken together, this work may provide a more theoretical basis for the development and utilization of ERP to improve gastric motility, promote intestinal health, and treat FD.

## 2 Materials and methods

### 2.1 Materials

The ELISA kits for Motilin (MTL) (H182-1-2), Gastrin (Gas) (H239-1-2), Cholecystokinin (CCK) (H160-1-2), Vasoactive Intestinal Peptide (VIP) (H219-1-2), and Calcitonin Gene-Related Peptide (CGRP) (H217-1-2) were purchased from Nanjing Jiancheng Bioengineering Institute (Nanjing, China). The Interleukin-6 (IL-6) kit (EK206), TNF-*α* kit (EK282), and NO kit (S0021) were obtained from Hangzhou Lianke Biotechnology Co., Ltd. All other chemical reagents were of analytical grade.

### 2.2 Preparation and structural characterization of ERP

The entire *Elsholtzia rugulosa* Hemsl. (Lamiaceae) plant was collected from Yunnan Province, China, in September 2022. The species identification was confirmed by Professor Zhu-Ya Yang of Yunnan University of Chinese Medicine. A voucher specimen (No. YBZ-202209) has been archived at the State Key Research Center of Nutrition and Health, Yunnan University of Chinese Medicine.

#### 2.2.1 Preparation of aqueous extract of *E. rugulosa* (AER)

The entire plant of *E. rugulosa* Hemsl. was placed in a medicinal separation bag and underwent two pre-treatment steps involving defatting with industrial methanol at 60 °C, each lasting for 3 h. The resulting filtrates were discarded, after which the dried herbal material was reintroduced into the medicinal separation bag and subjected to two water extractions at 90 °C, with each extraction also lasting for 3 h. Following filtration through gauze, the combined filtrates were concentrated and subjected to vacuum drying to yield the aqueous extract of *E. rugulosa* (AER). The resultant extract was subsequently stored at 4 °C for future use.

#### 2.2.2 Extraction and purification of *E. rugulosa* polysaccharides (ERP)

The extraction and purification of polysaccharides were performed in accordance with previously established protocols (16), with slight modifications. In summary, a concentrated solution was prepared by dissolving 400 grams of AER in pure water. Absolute ethanol was subsequently added to the solution to achieve an 80% alcohol concentration, with continuous agitation. Following a 24-h quiescent period, centrifugation was executed at 2000 revolutions per minute (rpm) for a duration of 10 min. Crude polysaccharides were extracted from *E. rugulosa* through precipitation. Subsequently, protein removal was conducted using the Sevage method. The volume of the concentrated solution was accurately determined, after which chloroform, constituting one-fifth of the concentrated solution’s volume, was added. This was followed by the addition of *n*-butanol in a volume equal to that of chloroform. The mixture was subjected to vigorous agitation for 30 min, followed by centrifugation at 2000 rpm for 10 min. The supernatant was collected, and the protein removal process was repeated five to eight times. The collected supernatants were then combined, concentrated under reduced pressure, vacuum-dried, and stored for potential future applications.

#### 2.2.3 Fractionation and purification of ERP (ERP-N and ERP-A)

A 2 g sample of ERP was dissolved in 10 mL of ultrapure water and subsequently centrifuged at 8000 rpm for 10 min using a ST16R centrifuge (Thermo Scientific, Germany) to eliminate insoluble components. The resulting solution was then subjected to ion exchange chromatography on a DEAE-Sepharose FF column, facilitated by a BT600 L peristaltic pump (Ke Jian, China) (17). The elution process was performed using a gradient series of NaCl solutions with molar concentrations of 0, 0.2, 0.4, 0.6, 0.8, and 1.0 mol/L, at a constant flow rate of 2.5 mL/min. The eluate was collected in 10 mL fractions using an automatic fraction collector (BSZ-100, Jinpeng Analytical Instruments Co., Ltd., Shanghai, China) and was subsequently analyzed by the phenol-sulfuric acid method. The collected eluate was then concentrated, subjected to dialysis with a molecular weight cut-off of 3,500 Da, and lyophilized. The resulting purified polysaccharides were designated as ERP-N (eluted with H_2_O) and ERP-A (eluted with 0.2 M NaCl).

### 2.3 Structural characterization of ERP-N and ERP-A

#### 2.3.1 Components analysis

The total carbohydrate content was quantified by the phenol–sulfuric acid method, with glucose serving as a standard. The total uronic acid content was assessed through *meta*-hydroxydiphenyl assay, employing glucuronic acid as a standard (18). Protein content was determined using Bradford’s method, with bovine serum albumin as a reference standard. Additionally, the endotoxin level in ERP-N and ERP-A were evaluated using an endpoint chromogenic Limulus amebocyte lysate assay, with lipopolysaccharide (LPS) employed as a positive control.

#### 2.3.2 Molecular weight determination

The weight-average molecular weights of ERP-N and ERP-A were ascertained utilizing a series of three tandem polymer matrix water-soluble size exclusion chromatography (SEC) columns (8 × 300 mm), integrated with a Waters high-performance liquid chromatography system (Waters 1,515, Waters, USA), maintained at a temperature of 40 °C. The weight-average molecular weights of ERP-N and ERP-A were determined using a calibration curve derived from dextran standards with varying weight-average molecular weights (1, 5, 12, 25, 50, 80, 150, 270, 410, and 670 kDa).

#### 2.3.3 Methylation and gas chromatography–mass spectrometry analysis

The methylation of ERP-N and ERP-A was conducted in accordance with established protocols. Subsequent to hydrolysis, reduction, and acetylation of the methylated samples, gas chromatography–mass spectrometry (GC–MS) analysis was performed on a GC–MS system. The mass spectrometry component consisted of a quadrupole mass detector (Agilent 5977B; Agilent Technologies, USA), while the chromatography was carried out using an Agilent gas chromatograph (Agilent 7890A; Agilent Technologies, USA), which was fitted with an HP-5MS capillary column (30 m × 0.25 mm × 0.25 *μ*m, Agilent J&W Scientific, Folsom, CA, USA). The methylation results were analyzed in accordance with established literature, leading to the identification of the linkage types of ERP-N and ERP-A. For uronic acid-containing residues, linkage assignments were interpreted cautiously and were further supported by monosaccharide composition, total uronic acid content, FT-IR, and NMR data, rather than relying solely on methylation analysis.

#### 2.3.4 Fourier transform infrared spectrometric analysis

Fourier Transform Infrared (FT-IR) spectroscopy of the polysaccharide samples was conducted on a Nicolet 6,700 spectrometer (Thermo Nicolet, USA). For the analysis, 2 mg of each polysaccharide sample was mixed with potassium bromide (KBr) powder and then compressed into 1-mm pellets. The FT-IR spectra were recorded over the spectral range of 4,000–400 cm^−1^.

#### 2.3.5 NMR spectroscopic analysis

Dried ERP-N and ERP-A (60 mg) were dissolved in 0.5 mL of D₂O. 1D NMR experiments, including ^1^H NMR, ^13^C NMR, and DEPT, as well as 2D NMR experiments, such as ^1^H-^1^H COSY, NOESY, HSQC, and HMBC, were recorded on a Bruker Ascend TM 800 MHz NMR spectrometer (Billerica, USA).

#### 2.3.6 Transmission electron microscopy (TEM) analysis

To investigate the microstructure of the polysaccharides ERP-N and ERP-A, TEM analysis was performed as described in a previous study. Briefly, the sample aqueous solution (1 mg/mL) was mixed with an equal volume of SDS solution (1 mg/mL). The mixture was then heated at 60 °C for 2 h and subsequently diluted with H_2_O to a final concentration of 5 μg/mL. Transmission electron microscopy (TEM, Talos L120C, Thermo Fisher Scientific, USA) with an accelerating voltage of 80 kV was applied to visualize the molecular morphology of the samples after drying.

### 2.4 Effects of AER and ERP on FD in rats

#### 2.4.1 Grouping, drug administration, and sample collection

According to previous studies (7), the gender of rats does not significantly influence the outcomes of the experiments. However, male rats were selected for the majority of these experiments to maintain consistency (19, 20). Therefore, male rats were selected for this experiment. Fifty-six healthy Sprague–Dawley (SD) rats, weighing between 180 and 220 grams, were obtained from SPF Biotech Co., Ltd. (Beijing) (Animal License Number: SCXK (Beijing) 2019–0010). These rats were housed in a Specific Pathogen Free (SPF) animal facility, where the ambient temperature was maintained at 24 ± 2 °C. They were provided with ad libitum access to food and water. Prior to the experimental procedures, the rats underwent a five-day acclimatization period under a controlled 12-h light–dark cycle. All animal experimental protocols were approved by the Animal and Ethics Review Committee of Kunming Quality Supervision and Inspection Institute (Kunming, Yunnan, China) in accordance with the National Guidelines for the Welfare of Laboratory Animals (Ethics Approval Number: R-062024010).

#### 2.4.2 Animals and drug treatment

The FD models were developed in all experimental groups, with the exception of the Control group (*n* = 7), utilizing established methods such as the classic tail-pinch, irregular feeding, and exposure to ice saline (21, 22). Hemostatic forceps, wrapped in gauze, were employed to clamp the proximal third of the tail, followed by immediate release to induce aggressive behavior in the rats for a duration of 30 min, conducted twice daily at 9 a.m. and 1 p.m. In instances where skin lesions resulted from clamping or aggressive interactions, the affected skin areas were treated with 2% iodophor to prevent secondary infection. The animals underwent fasting on Wednesdays, Fridays, and Sundays, and were subjected to tail pinching and irregular feeding schedules over a period of 3 weeks. Upon necropsy, no organic lesions were observed in the gastric and intestinal tissues, thereby confirming the successful establishment of the model and the fulfillment of the diagnostic criteria for FD. Subsequently, 49 rats were randomly allocated into the following groups: the model group (*n* = 7), the domperidone group (*n* = 6, 4 mg/kg), the AER-L group (*n* = 6, 116 mg/kg), the AER-M group (*n* = 6, 223 mg/kg), the AER-H group (*n* = 6, 666 mg/kg), the ERP-L group (*n* = 6, 25 mg/kg), the ERP-M group (*n* = 6, 50 mg/kg), and the ERP-H group (*n* = 6, 100 mg/kg). Following successful modeling, the respective drugs were administered once daily over a 14-days treatment period. After the final administration, all Sprague–Dawley rats were subjected to a 16-h fasting period, during which fecal samples were collected. Subsequently, the subjects were anesthetized and euthanized. Blood samples were collected and centrifuged at 4000 rpm for 15 min at 4 °C to separate the serum, which was then stored in an ultra-low temperature freezer prior to analysis. Tissue samples, including those from the stomach, spleen, duodenum, and cecal contents, were obtained and preserved at −80 °C. Portions of the ileum tissue and gastric antrum were immersed in a 4% paraformaldehyde phosphate-buffered solution for further processing.

### 2.5 Histological analysis

A portion of the gastric antrum and duodenum was preserved in a 4% paraformaldehyde solution for 24 h, subsequently embedded in paraffin, and sectioned into consecutive slices with a thickness of 5 *μ*m. These sections were stained using hematoxylin and eosin (H&E) and underwent standard histopathological analysis under a microscope.

### 2.6 Enzyme-linked immunosorbent assay (ELISA)

The aliquoted serum samples were collected, and the concentrations of MTL, GAS, CCK, VIP, and CGRP were quantified by ELISA kits. All experimental procedures adhered to the protocols outlined by the manufacturer.

### 2.7 Gut microbiota analysis

The 16S rRNA sequencing of bacteria in intestinal content samples was conducted by Servicebio Technology Co., Ltd., Wuhan, China. DNA was extracted from sterile samples using the T Guide S96 magnetic bead-based soil/fecal genomic DNA extraction kit (DPDP812, Tiangen Biotech (Beijing) Co., Ltd., China). After electrophoresis inspection, PCR amplification was performed. The obtained DNA was used to target the V4 region of the 16S rRNA gene with forward primer 515F (5’-GTGYCAGCM-GCCGCGGTAA-3′) and reverse primer 806R (5’-GGACTACNVGG-GTWTCTAAT-3′). The PCR products were detected using 1.8% agarose gel electrophoresis. The amplification results were sequenced using the Illumina Novaseq 6,000 platform with PE250 sequencing analysis.

### 2.8 Transcriptome analysis

Total RNA was extracted from gastric tissue and purified using TRIzol reagent (QIAzol Lysis Reagent, Germany) according to the manufacturer’s instructions. Library preparation focused on extracting total RNA, enriching mRNA, reverse transcribing into cDNA, adapter ligation, and Illumina sequencing. The software fastp was used to remove reads containing sequencing adapters, low-quality reads, and reads with an unknown base ratio >10% to obtain clean reads. The software DEGseq2 was used for differential expression analysis, with the default screening criteria for significantly differentially expressed genes (DEGs) being FDR < 0.05 & |log2FC| ≥ 1. A gene was considered a DEG if it met both criteria simultaneously. Finally, the software Goatools was used for GO enrichment analysis of DEGs, and the software KOBAS was used for KEGG pathway enrichment analysis of DEGs. Library preparation and Illumina sequencing were performed at Majorbio (Shanghai, China).

### 2.9 Western blot analysis

The total proteins were extracted from rat’s gastric tissues. After being quantified by BCA kits, an equivalent amount of proteins was separated by SDS-PAGE. The proteins were transferred to PVDF membrane, blocked with 5% skim milk dissolved in TBST, and then incubated overnight at 4 °C with the following primary antibodies targeting PI3k, Akt, PKG, and *β*-actin. Followed by HRP-conjugated secondary antibody incubations, chemical imaging was performed with ultrasensitive ECL chemiluminescence reagent. Band intensities were quantified using Image Pro 6.0 software (Media Cybernetics, Baltimore, MD, USA).

### 2.10 Immunomodulatory activity evaluation

RAW 264.7 cells were purchased from the Medical College of Sun Yat-Sen University and cultured according to our previously reported method (23). Cell viability was determined using Cell Counting Kit-8 (CCK-8; Absin Bioscience Inc., China). For immunomodulatory activity assay, RAW 264.7 cells (1 × 10^5^ cells/100 *μ*L) were pre-stimulated with 10 μg/mL of LPS for 3 h, and then the LPS-activated cells were treated with 10, 25, 100, and 200 μg/mL of ERP-N and ERP-A. After 24 h of incubation, the cell supernatants were collected for NO, IL-6, and TNF-*α* determination.

### 2.11 Statistical analysis

The results of this study were analyzed using SPSS 19.0 software. The results are presented as mean ± standard deviation (mean ± S). One-way analysis of variance (ANOVA) was used for statistical analysis. *p* < 0.05 was considered to be statistically significant.

## 3 Results

### 3.1 Preparation and chemical composition

**Figure 1:**
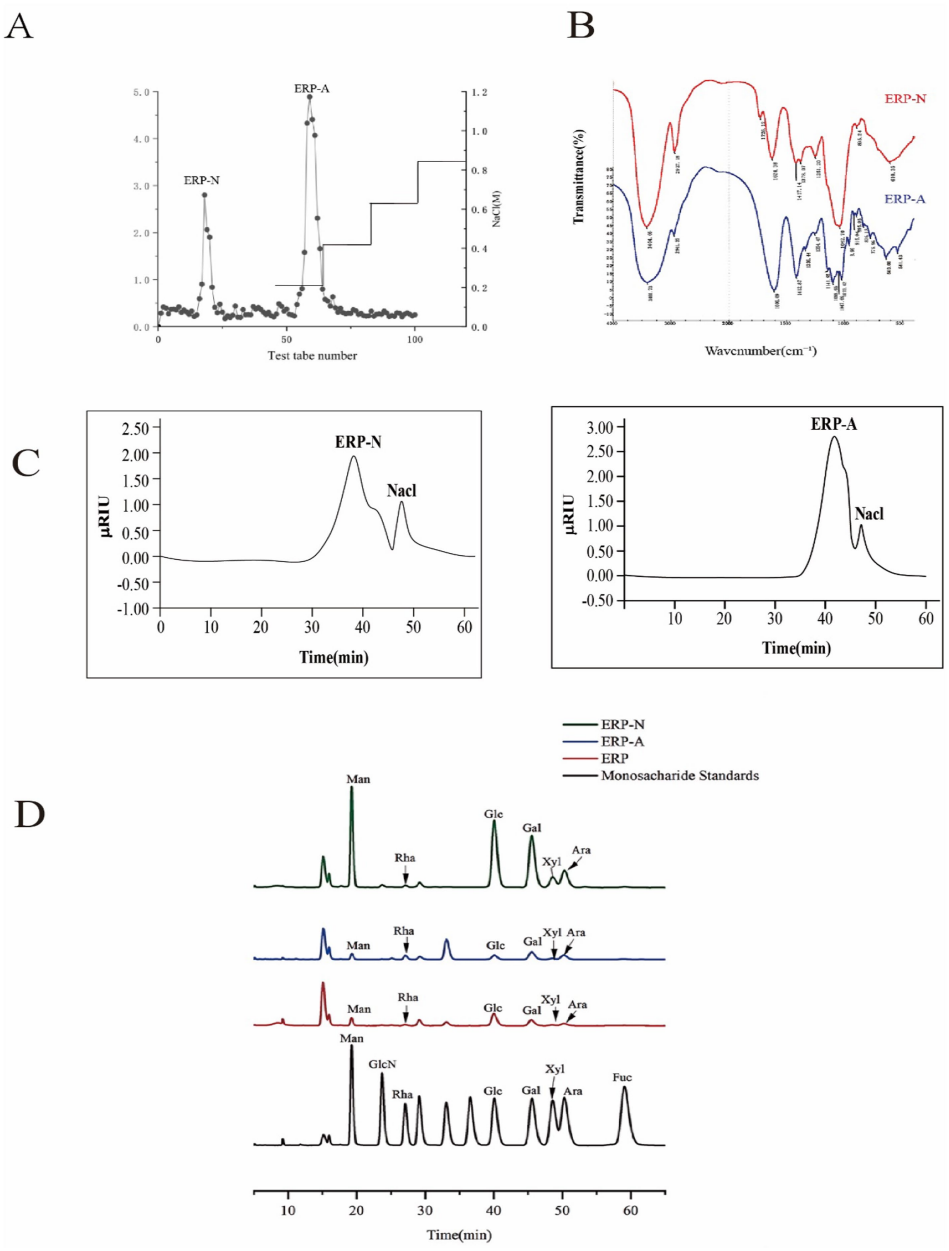
Characterization of the physical and chemical properties of ERP-N and ERP-A. **(A)** HPGPC chromatogram analysis of ERP-N and ERP-A; **(B)** Fourier transform infrared spectrum of ERP-N and ERP-A; **(C)** HPGPC chromatograms of ERP-N and ERP-A; **(D)** Chromatogram of the monosaccharide composition for ERP, ERP-N, and ERP-A.

The novel polysaccharides were extracted from the Lamiaceae family employing a sequential methodology that included methanol degreasing, aqueous extraction, ethanol precipitation, DEAE-Sepharose FF ion exchange chromatography, and Sephadex G-100 gel filtration chromatography (refer to Figure 1A). The polysaccharides were designated as ERP-N and ERP-A. The molecular weight analysis performed using high-performance gel permeation chromatography (HPGPC) showed that ERP-N and ERP-A each exhibited a single major symmetrical peak in the chromatograms (Figure 1C), indicating relatively uniform molecular weight distributions. As shown in Table 1, the peak area percentages of ERP-N and ERP-A were both 100%, with distribution coefficients (Mw/Mn) of 1.42 and 1.33, respectively. These results suggest that ERP-N and ERP-A were purified polysaccharide fractions with good homogeneity under the current chromatographic conditions. The weight-average molecular weights of ERP-N and ERP-A were 13,415 Da and 3,691 Da, respectively. ERP-N consisted of mannose, rhamnose, glucose, galactose, xylose, and arabinose, with molar percentages of 20.38, 1.81, 32.85, 23.59, 8.27, and 9.99%, respectively, as depicted in Figure 1D. Conversely, ERP-A was composed of mannose, rhamnose, glucose, galactose, xylose, and arabinose, with respective molar percentages of 5.49, 14.04, 9.31, 14.52, and 4%, as depicted in Figure 1D.

**Table 1:** Results of molecular weight and distribution for ERP-N and ERP-A.

Polysaccharide component	RT (min)	Mp (Da)	Mw (Da)	Mn (Da)	Peak area percentage (%)	Distribution coefficient (MW/Mn)
ERP-N	38.654	11,286	13,415	9,458	100	1.42
ERP-A	41.792	3,302	3,691	2,775	100	1.33

The FT-IR spectra showed that ERP-N and ERP-A exhibited the characteristic absorptions of the polysaccharides at 3400.73 cm^−1^ and 3404.65 cm^−1^, 2941.35 cm^−1^ and 2927.18 cm^−1^,1605.09 cm^−1^, 1725.12 cm^−1^, and 1624.38 cm^−1^ for O–H, C–H, and C–O stretching vibrations, respectively (Figure 1B). The absorptions at 1000–1200 cm^−1^ were ascribed to C–O–C and C–O–H in a pyranose ring. The bands at 958.96 cm^−1^ and 915.04 cm^−1^ were characteristic of *β*-glycosidic linkages. Furthermore, the bands at 895.06 cm^−1^ and 895.24 cm^−1^, which arise from the *β*-anomeric C–H, were indicative of a *β*-pyranose structure. The absorption band at 836.13 cm^−1^ corresponded to the wagging vibration of the C–H group in the anomeric position of *α*-pyranose. Lastly, the peak at 775.96 cm^−1^ represented the symmetric ring stretching vibration of the pyran ring.

**Figure 2:**
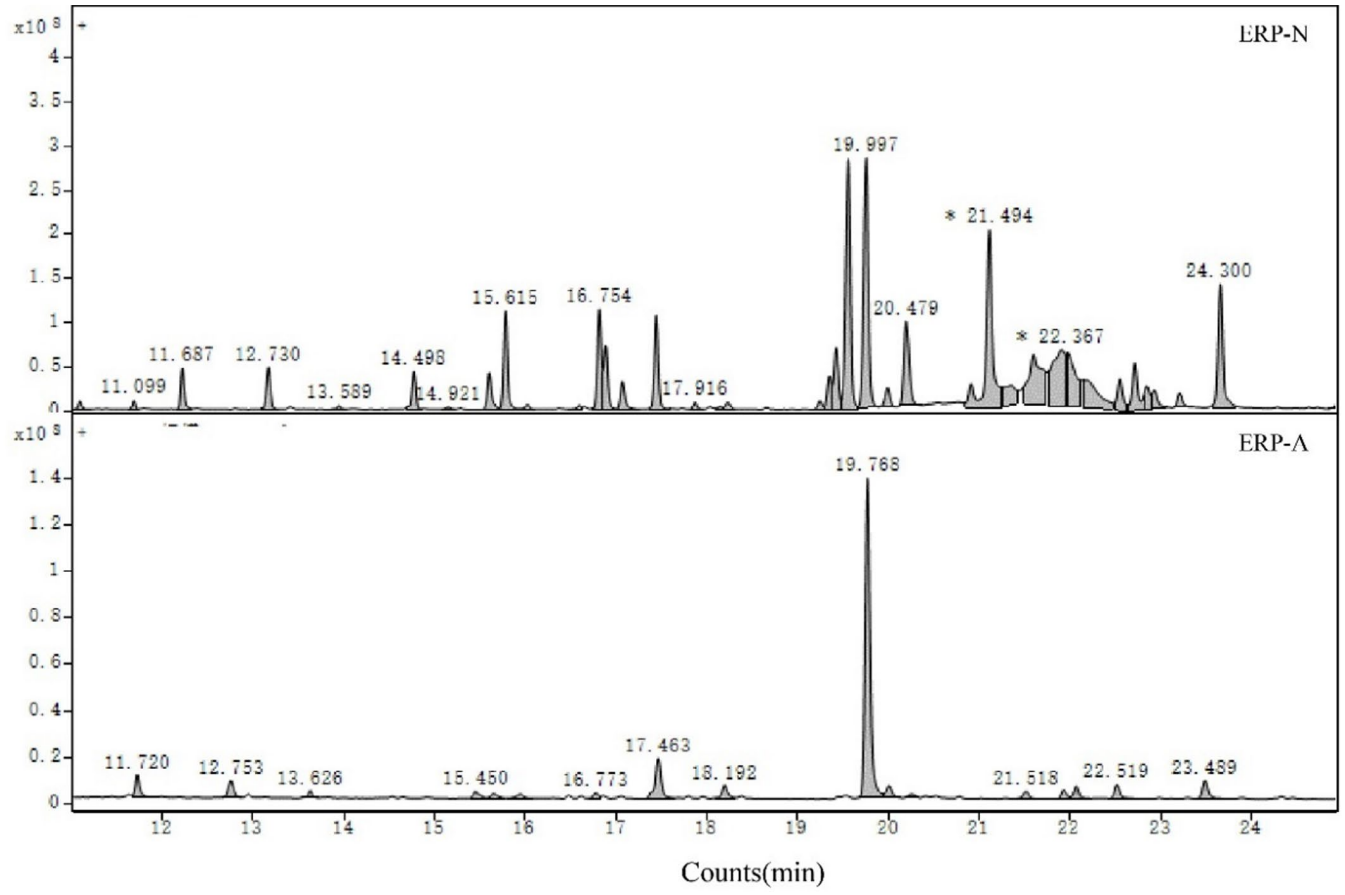
Analysis chart of linkage types for ERP-N and ERP-A.

The glycosidic linkages in ERP-N and ERP-A were elucidated via methylation analysis. The identification of the PMAA derivatives of ERP-N and ERP-A was achieved by comparing their retention times and mass fragments (Figure 2) with reference data from the CCRC Spectral Database for PMAAs. As presented in Table 2, the analysis reveals that ERP-N and ERP-A predominantly comprise six monosaccharide derivatives, corroborating the results obtained from the monosaccharide composition analysis. Specifically, ERP-N’s major derivatives include 1,5,6-tri-*O*-acetyl-2,3,4-tri-*O*-methyl galactitol, 1,4,5-tri-*O*-acetyl-2,3,6-tri-*O*-methyl galactitol, 1,4,5-tri-*O*-acetyl-2,3,6-tri-*O*-methyl glucitol, and 1,3,5,6-tetra-*O*-acetyl-2,4-di-*O*-methyl galactitol. These derivatives exhibited primary linkage patterns of 6-Gal(p), 4-Gal(p), and 4-Glc(p). Considering the methylation results together with uronic acid content, FT-IR characteristics, and NMR assignments, the predominant GalA-containing linkage patterns in ERP-A were inferred to be mainly →4)-GalA(p) and terminal GalA(p)-related residues.

**Table 2:** Linkage types of monosaccharides, derivative names, and relative molar ratios in ERP-N.

Type of linkage	Glycosidic linkage	Derivative names	RT (min)	Relative molar ratio (100%)
Gal	t-Gal(f)	1,4-di-O-acetyl-2,3,5,6-tetra-O-methyl galactitol	17.034	1.63
	t-Gal(p)	1,5-di-O-acetyl-2,3,4,6-tetra-O-methyl galactitol	17.447	5.06
	4-Gal(p)	1,4,5-tri-O-acetyl-2,3,6-tri-O-methyl galactitol	19.781	14.53
	3-Gal(p)	1,3,5-tri-O-acetyl-2,4,6-tri-O-methyl galactitol	20.259	1.07
	6-Gal(p)	1,5,6-tri-O-acetyl-2,3,4-tri-O-methyl galactitol	21.494	14.1
	4,6-Gal(p)	1,4,5,6-tetra-O-acetyl-2,3-di-O-methyl galactitol	23.502	0.93
	3,6-Gal(p)	1,3,5,6-tetra-O-acetyl-2,4-di-O-methyl galactitol	24.3	6.72
Glc	t-Glc(p)	1,5-di-O-acetyl-2,3,4,6-tetra-O-methyl glucitol	16.754	5.06
	2-Glc(p)	1,2,5-tri-O-acetyl-3,4,6-tri-O-methyl glucitol	19.435	0.45
	3-Glc(p)	1,3,5-tri-O-acetyl-2,4,6-tri-O-methyl glucitol	19.634	3.4
	4-Glc(p)	1,4,5-tri-O-acetyl-2,3,6-tri-O-methyl glucitol	19.997	13.81
	6-Glc(p)	1,5,6-tri-O-acetyl-2,3,4-tri-O-methyl glucitol	20.479	5.18
	4,6-Glc(p)	1,4,5,6-tetra-O-acetyl-2,3-di-O-methyl glucitol	23.258	3
Ara	t-Ara(f)	1,4-di-O-acetyl-2,3,5-tri-O-methyl arabinitol	11.687	2.17
	3-Ara(f)	1,3,4-tri-O-acetyl-2,5-di-O-methyl arabinitol	14.498	2.04
	5-Ara(f)	1,4,5-tri-O-acetyl-2,3-di-O-methyl arabinitol	15.412	2.16
	3,5-Ara(f)	1,3,4,5-tetra-O-acetyl-2-O-methyl arabinitol	17.916	0.34
	2,5-Ara(f)	1,2,4,5-tetra-*O*-acetyl-3-*O*-methyl arabinitol	18.311	0.44
Xyl	t-Xyl(p)	1,5-di-O-acetyl-2,3,4-tri-O-methyl xylitol	12.73	2.55
	2-Xyl(p)	1,2,5-tri-O-acetyl-3,4-di-O-methyl xylitol	15.615	1.43
	4-Xyl(p)	1,4,5-tri-O-acetyl-2,3-di-O-methyl xylitol	15.615	4
	3,4-Xyl(p)	1,3,4,5-tetra-O-acetyl-2-O-methyl xylitol	18.219	0.16
Man	3,6-Man(p)	1,3,5,6-tetra-O-acetyl-2,4-di-O-methyl mannitol	23.805	0.74
	2,6-Man(p)	1,2,5,6-tetra-O-acetyl-3,4-di-O-methyl mannitol	23.405	1.5
	4,6-Man(p)	1,4,5,6-tetra-O-acetyl-2,3-di-O-methyl mannitol	23.079	1.87
	2-Man(p)	1,2,5-tri-O-acetyl-3,4,6-tri-O-methyl mannitol	19.551	1.73
	t-Man(p)	1,5-di-O-acetyl-2,3,4,6-tetra-O-methyl mannitol	16.832	3.36
Fuc	t-Fuc(p)	1,5-di-O-acetyl-6-deoxy-2,3,4-tri-O-methyl fucitol	13.589	0.25
	4-Fuc(p)	1,4,5-tri-O-acetyl-6-deoxy-2,3-di-O-methyl fucitol	15.881	0.33

**Figure 3:**
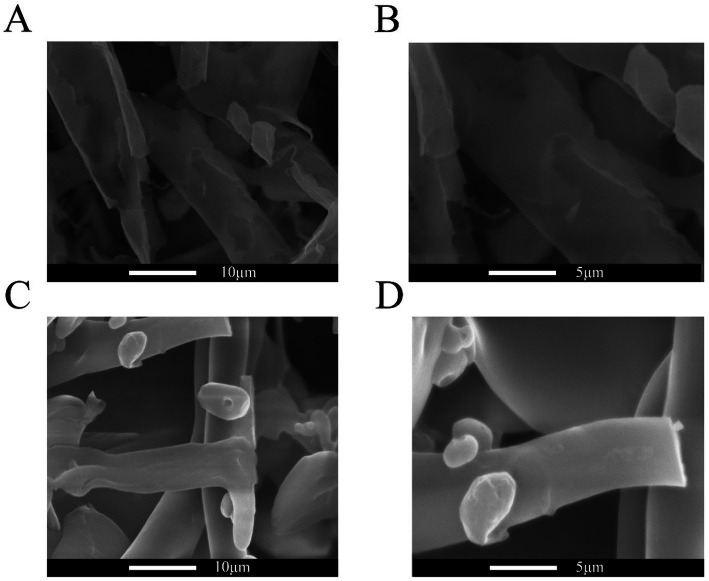
TEM images of ERP-N and ERP-A in sodium dodecyl sulfate solution. **(A,B)** TEM images of ERP-N (5,000×,10,000×); **(C,D)** TEM images of ERP-A (5,000×,10,000×).

The TEM images depicting ERP-N and ERP-A are presented in Figure 3. Specifically, ERP-N molecules exhibited a propensity to adhere to the crystals in a platelet-like configuration, as illustrated in Figures 3A,B. Conversely, ERP-A displayed rhamnose moieties attached in a vesicular morphology, as evidenced in Figures 3C,D. These observations align coherently with the findings obtained from GC–MS and NMR analyses.

**Figure 4:**
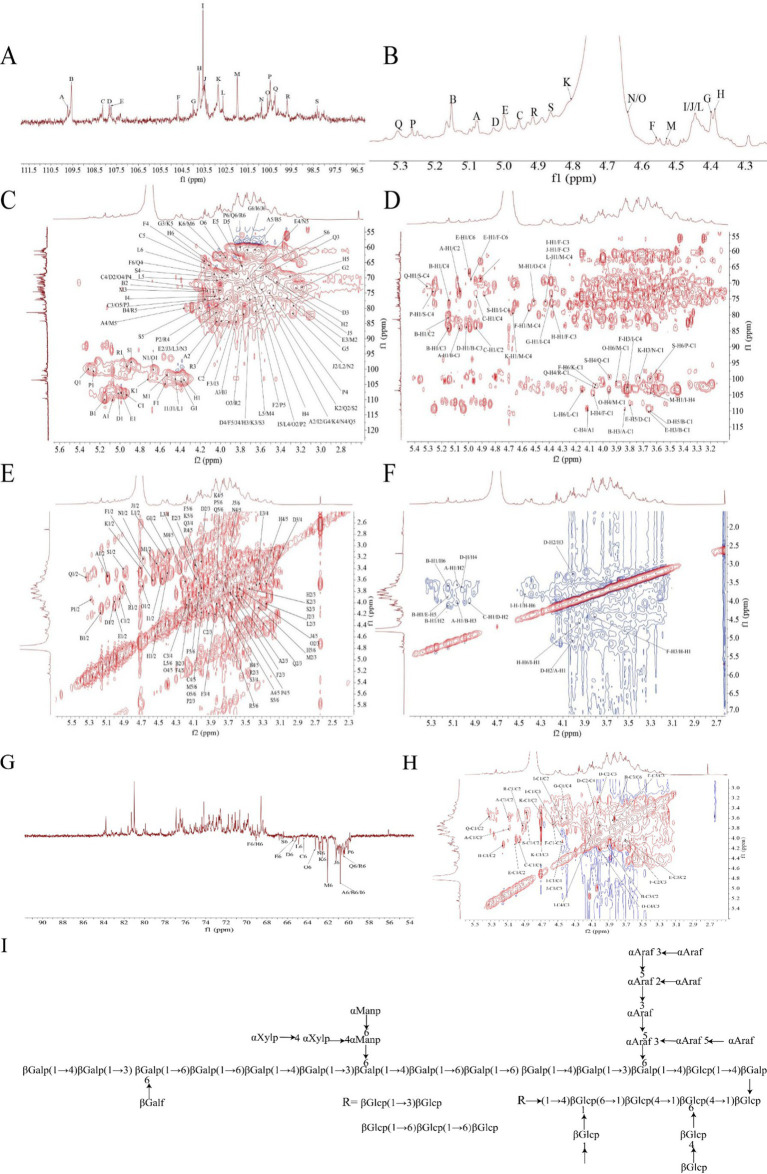
ERP-N NMR maps. **(A)** carbon spectrum (^13^C NMR). **(B)** One-dimensional hydrogen ^1^H NMR). **(C)** Hydrogen–hydrogen correlation spectrum (^1^H-^1^H COSY). **(D)** Nuclear Overhauser Effect spectrum (NOESY). **(E)** Heteronuclear Single Quantum Coherence spectrum (HSQC). **(F)** Heteronuclear Multiple Bond Correlation (HMBC) spectrum. **(G)** ^13^C NMR-DEPT 135. **(H)** Total Correlation spectrum (TOCSY). **(I)** The putative structure of ERP-N.

**Figure 5:**
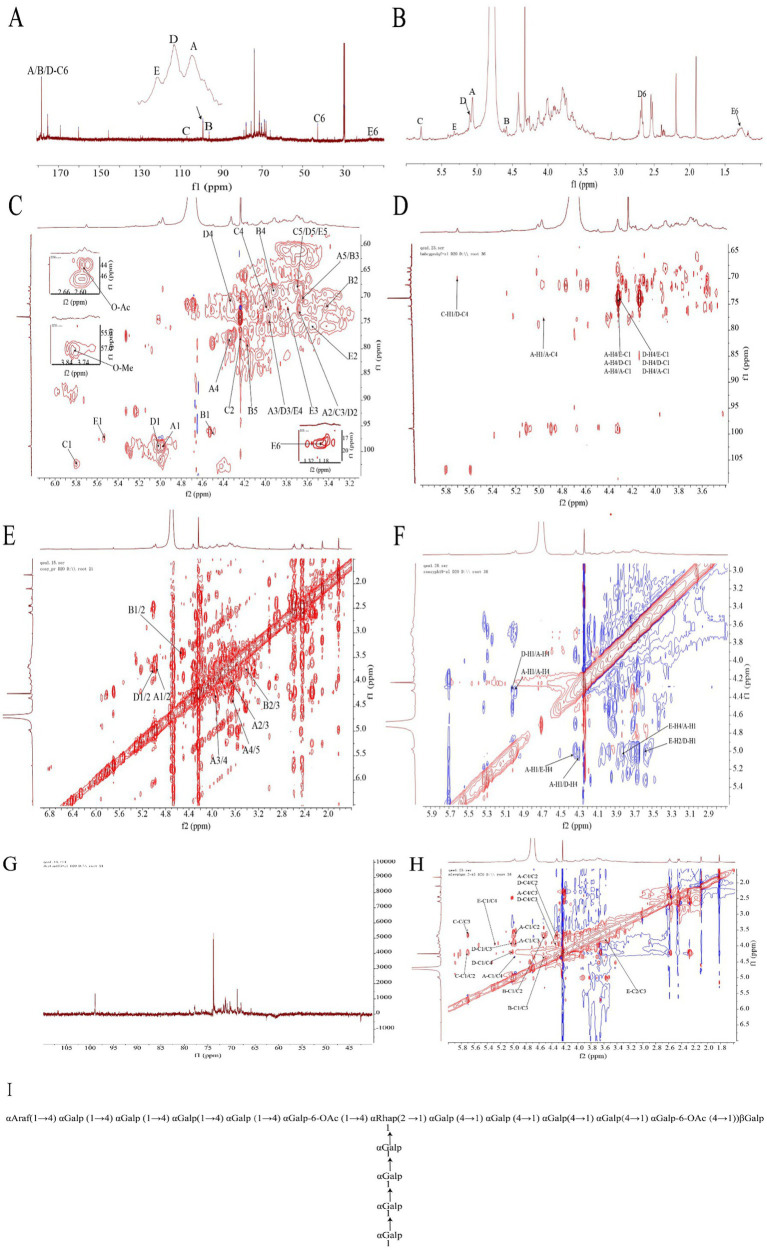
ERP-A NMR maps. **(A)** carbon spectrum (^13^C NMR). **(B)** One-dimensional hydrogen (^1^H NMR). **(C)** Hydrogen–hydrogen correlation spectrum (^1^H-^1^H COSY). **(D)** Nuclear Overhauser Effect spectrum (NOESY). **(E)** Heteronuclear Single Quantum Coherence spectrum (HSQC). **(F)** Heteronuclear Multiple Bond correlation spectrum (HMBC). **(G)** ^13^C NMR-DEPT 135. **(H)** Total correlation spectrum (TOCSY). **(I)** The putative structure of ERP-A.

To further elucidate the glycosidic linkage configurations of ERP-N and ERP-A, one-dimensional proton nuclear magnetic resonance (^1^H-NMR) spectroscopy was employed. The proton signals of polysaccharides predominantly resided within the chemical shift range of *δ* 3.0–5.5 ppm, with the anomeric proton (H-1) resonance region typically spanning from δ 4.5 to 5.5 ppm (24, 25). The ^1^H-NMR spectra of the polysaccharide samples (Figures 4B,E, 5B,E) exhibited a dense concentration of proton resonance signals within this range, resulting in significant signal overlap. Multiple proton signals were identified within the anomeric region, while other proton signals were concentrated in the δ 4.5–3.0 ppm region, also experiencing severe overlap, thereby challenging assignment. The chemical shifts of anomeric carbon (C-1) signals for polysaccharides generally fall between δ 90 and 110 ppm (Figures 4A, 5A). Specifically, *α*-configured anomeric carbon signals typically appear between δ 95 and 103 ppm, whereas *β*-configured anomeric carbon signals are usually observed above δ 101 ppm (Figures 4G, 5G). The signals for C-2 to C-5 cluster in the δ 65–85 ppm region. The chemical shifts of substituted carbon atoms undergo glycosylation shifts, moving towards the downfield region (26, 27); see Table 3.

**Table 3:** linkage types of monosaccharides, derivative names, and relative molar ratios in ERP-A.

Type of linkage	Glycosidic linkage	Derivative names	RT (min)	Relative molar ratio (100%)
Gal	6-Gal(p)	1,5,6-tri-O-acetyl-2,3,4-tri-O-methyl galactitol	21.518	1.6
	3,4-GalA(p)	1,3,4,5-tetra-O-acetyl-2,6-di-O-methyl galactitol	21.927	1.54
	2,4-GalA(p)	1,2,4,5-tetra-O-acetyl-3,6-di-O-methyl galactitol	22.519	2.74
	4,6-GalA(p)	1,4,5,6-tetra-O-acetyl-2,3-di-O-methyl galactitol	23.489	3.81
	t-GalA(p)	1,5-di-O-acetyl-2,3,4,6-tetra-O-methyl galactitol	17.463	10.51
	4-GalA(p)	1,4,5-tri-O-acetyl-2,3,6-tri-O-methyl galactitol	19.768	61.91
Glc	t-Glc(p)	1,5-di-O-acetyl-2,3,4,6-tetra-O-methyl glucitol	16.773	1.21
	4-Glc(p)	1,4,5-tri-O-acetyl-2,3,6-tri-O-methyl glucitol	20.007	2.86
Ara	t-Ara(f)	1,4-di-O-acetyl-2,3,5-tri-O-methyl arabinitol	11.72	4.76
	5-Ara(f)	1,4,5-tri-O-acetyl-2,3-di-O-methyl arabinitol	15.45	1.83
Xyl	4-Xyl(p)	1,4,5-tri-O-acetyl-2,3-di-O-methyl xylitol	15.647	1.33
Fuc	t-Fuc(p)	1,5-di-O-acetyl-6-deoxy-2,3,4-tri-O-methyl fucitol	13.626	1.61
Rha	t-Rha(p)	1,5-di-O-acetyl-6-deoxy-2,3,4-tri-O-methyl rhamnitol	12.753	4.29

To obtain detailed structural information on ERP-N and ERP-A, a comprehensive suite of NMR techniques was conducted, including ^1^H-NMR, ^13^C-NMR, DEPT135, and two-dimensional NMR methods such as Heteronuclear Single Quantum Coherence (HSQC), Heteronuclear Multiple Bond Correlation (HMBC), Proton-Proton Correlation Spectroscopy (^1^H-^1^H COSY), Nuclear Overhauser Effect Spectroscopy (NOESY), and Total Correlation Spectroscopy (TOCSY). These measurements provided exhaustive H and C chemical shift data for each major sugar residue, enabling the deduction of the linkage sequence among sugar residues. Based on the monosaccharide composition and methylation analysis results of the polysaccharide samples, analysis of the NMR spectra revealed that ERP-N exhibited 19 distinct anomeric proton and anomeric carbon signals, suitable for structural analysis (16, 24, 26, 28–46). These anomeric regions exhibited specific chemical shifts at distinct *δ* ppm values (Table 4), along with reducing end signals at δ 5.07/109.4 ppm, δ 4.39/103.6 ppm, δ 4.44/103.1 ppm, δ 4.66/100.6 ppm, δ 5.25/100.1 ppm, and δ 4.91/99.4 ppm, designated as A, B, C, D, E, F, G, H, I, J, K, L, M, N, O, P, Q, R, and S, respectively. After assigning the anomeric signals, the ^1^H and ^13^C chemical shift signals of the primary sugar residue types in the polysaccharide samples were attributed through ^1^H-^1^H COSY, HSQC, HMBC, NOESY, and TOCSY, in conjunction with ^1^H-NMR and ^13^C-NMR spectra and methylation results, and by referencing chemical shift data for similarly substituted sugar residues reported in relevant literature. The results are summarized in Table 4. For sugar residues A-E, at *δ* 5.07/109.4 ppm, δ 5.15/109.2 ppm, δ 4.95/107.8 ppm, δ 5.02/107.5 ppm, and δ 4.99/107.4 ppm, the anomeric correlations of *α*-L-Ara(f) were assigned to non-reducing termini (residue A), (→3)-linked (B), (→5)-linked (C), (→2,5)-linked (D), and (→3,5)-linked α-L-Ara(f) units (E), respectively (28, 29, 31, 32, 36, 38, 43, 46) Despite overlapping coupling networks, meticulous characterization using ^1^H-^1^H COSY allowed for the determination of the chemical shifts of all protons in A-E. TOCSY (Table 5 and Figure 4H) and NOESY spectra (Table 5 and Figure 4F) were also acquired. By comparing HSQC (Figure 4C) and HMBC (Figure 4D) spectra with literature references, corresponding carbons and their possible linkages could be inferred. Based on methylation results, the presence of side chains was hypothesized (Figure 4I). Similar methodologies were utilized to deduce the hydrogen and carbon signals of other major residues, with the assignments of major monosaccharide residue H and C chemical shifts in ERP-N presented in Table 4, Wang et al. (10), Yuhong and Fengshan (25), Ming et al. (27), Fu et al. (30), Guo et al. (32), Mou et al. (34); Liang et al. (47). Combining methylation results with literature reports, F-S were deduced as shown in Table 4. Among the assigned residues, residue G was assigned as terminal *β*-D-Galf based on combined methylation and NMR evidence. Unlike uronic acid-containing residues, Galf is a neutral sugar residue, for which conventional methylation analysis can provide useful linkage information. The detection of the terminal galactofuranose-related derivative 1,4-di-O-acetyl-2,3,5,6-tetra-O-methyl galactitol supported the presence of a terminal Galf residue. In the HSQC spectrum, residue G showed an anomeric H-1/C-1 correlation at δ 4.39/103.6 ppm, which was consistent with reported β-D-galactofuranose residues. In addition, the HMBC correlation from H-1 of residue G to C-4 of residue I further supported the possible linkage of terminal β-D-Galf to residue I. However, the signals of the remaining sugar residues were not clearly detected in the ^1^H NMR spectra of ERP-N and ERP-A, which may be attributed to the low content of these residues (28, 30).

**Table 4:** ^**1**^H and ^**13**^C NMR assignments for ERP-N and ERP-A.

Fraction	Residues		H1/C1	H2/C2	H3/C3	H4/C4	H5/C5	H6/C6	OCH_3_	COOH
ERP-N	A	T-α-L-Araf	5.07/109.4	3.61/72.96	3.84/81.7	4.23/72.07	3.66/3.74/61.1			
	B	1,3-α-L-Araf	5.15/109.2	4.12/80.53	3.84/83.61	4.02/76.7	3.66/3.74/61.1			
	C	1,5-α-L-Araf	4.95/107.8	3.92/83.46	4.14/76.98	4.01/73.2	4.12/65.86			
	D	1,2,5-α-L-Araf	5.02/107.5	4.0/74.56	3.27/73.32	3.68/76.42	3.66/3.80/66.97			
	E	1,3,5-α-L-Araf	4.99/107.4	4.05/81.4	3.67/76.9	3.73/71.41	3.80/66.75			
	F	1,3,6-β-D-Galp	4.55/104.4	3.66/75.89	3.82/77.3	4.12/68.9	3.70/76.89	3.94/4.05/69.1		
	G	T-β-D-Galf	4.39/103.6	3.20/73.23	3.87/69.52	3.60/73.64	3.28/78.49	/		
	H	1,6-β-D-Galp	4.38/103.34	3.45/73.1	3.73/75.9	3.66/72.57	3.33/68.95	3.94/4.05/69.1		
	I	1,4-β-D-Galp	4.44/103.2	3.56/72.43	3.87/80.3	4.06/74.07	3.61/78.39	3.73/61.2		
	J	T-β-D-Galp	4.44/103.1	3.28/81.4	4.04/80.05	3.70/77.0	3.55/69.8	3.73/61.55		
	K	1,3-β-D-Glc	4.70/102.4	3.46/78.8	3.74/74.76	3.60/72.87	3.84/69.8	4.01/62.94		
	L	1,6-β-D-Glcp	4.44/102.2	3.28/81.4	4.04/80.44	3.65/72.2	4.0/69.8	4.15/66.41		
	M	1,4-β-D-Glcp	4.52/101.6	3.36/75.47	4.06/73.66	3.64/76.76	4.24/72.27	4.01/62.47		
	N	T-β-D-Glcp	4.66/100.6	3.26/81.78	4.02/80.43	3.75/70.23	/	4.01/63.20		
	O	1,4,6-β-D-Glcp	4.66/100.2	3.55/76.8	3.70/80.43	3.96/74.15	4.15/77.01	3.91/63.93		
	P	T-α-D-Manp	5.25/100.1	3.92/76.94	4.15/77.01	4.00/74.15	3.66/75.90	3.82/60.7		
	Q	1,4-α-D-Xylp	5.30/99.9	3.54/77.38	3.83/73.86	4.00/69.8	3.62/72.89	3.82/60.86		
	R	T-α-D-Xylp	4.91/99.4	3.76/81.3	4.0/83.5	3.91/77.99	4.01/76.68	3.82/60.86		
	S	1,4,6-α-D-Manp	4.89/98.01	3.45/78.46	3.73/75.2	3.96/70.67	4.21/78.95	3.66/67.12		
ERP-A	A	→4)-α-GalpA-(1→	4.98/99.3	3.70/71.31	3.93/73.87	4.32/77.7	3.66/70.30	/178.5		2.58
	B	β-GalpA(1→	4.52/96.4	3.43/71.27	3.65/70.2	3.91/68.7	4.17/78.4	/178.5		2.58
	C	α-Araf-(1→	5.69/107.1	4.24/77.8	3.67/71.27	4.00/71.3	3.71/4.33/68.2	/		
	D	→4)-α-GalpA-6-O-Me-(1→	5.02/99.2	3.73/71.31	3.93/73.87	4.31/70.3	3.70/68.2	/169.2	3.80	
	E	→2,4)-α-Rhap-(1→	5.30/99.08	3.56/75.39	3.79/71.30	3.92/73.9	3.70/68.2	1.20/17.1		

**Table 5:** Major proton and carbon correlations observed in HMBC, NOESY, and TOCSY spectra of ERP-N and ERP-A (δ in ppm).

Fraction	Residues	δ_H_	Observed connections					
			HMBC		TOCSY		NOESY	
			δ_C_	Atom (sequence)	δ_H_	Atom	δ_H_	Atom
	T-α-L-Araf	5.07(A, H-1)	72.9	C-2 of residue A	3.61	H-2 of residue A	3.84	H-3 of residue B(B → A)
			81.7	C-3 of residue A	3.84	H-3 of residue A	/	
			83.6	C-3 of residue B(B → A)			/	
	1,3-α-L-Araf	5.15(B, H-1)	76.7	C-4 of residue B	3.84	H-3 of residue B	3.7	H-5 of residue E(E → B)
			80.5	C-2 of residue B	4.12	H-2 of residue B	3.84	H-3 of residue B
			83.6	C-3 of residue B	/		4.12	H-2 of residue B
		3.84(B, H-3)	109.4	C-1 of residue A(A → B)	4.12	H-2 of residue B	/	
	1,5-α-L-Araf	4.95(C, H-1)	73.2	C-4 of residue C	4.01	H-4 of residue C	4	H-2 of residue D(D → C)
		4.12(C, H-5)	109.4	C-1 of residue A(A → C)	/		/	
	1,2,5-α-L-Araf	5.02(D, H-1)	83.6	C-3 of residue B(B → D)	/		3.68	H-2 of residue D
		4.00(D, H-2)	/		3.27	H-3 of residue D	3.68	H-4 of residue D
			/		3.68	H-4 of residue D	5.07	H-1 of residue A(A → D)
		3.66(D, H-5)	109.2	C-1 of residue B(B → D)	/		/	
	1,3,5-α-L-Araf	4.99(E, H-1)	66.8	C-6 of residue E	4.05	H-2 of residue E	3.7	H-5 of residue E
			69.1	C-6 of residue F(F → E)	/		4.05	H-2 of residue E
			76.9	C-3 of residue E	/		/	
			81.4	C-2 of residue E	/		/	
		3.67(E, H-3)	109.2	C-1 of residue B(B → E)	4.05	H-2 of residue E	/	
		3.80(E, H-5)	107.5	C-1 of residue D(D → E)	3.67	H-3 of residue E	/	
	1,3,6-β-D-Galp	4.55(F, H-1)	76.7	C-4 of residue M(M → F)	3.66	H-2 of residue F	3.6	H-4 of residue F
					/		3.82	H-3 of residue F
		3.82(F, H-3)	103.2	C-1 of residue I(I → F)	/		4.38	H-1 of residue H(H → F)
		4.05(F, H-6)	102.4	C-1 of residue K(K → F)	/		/	
	T-β-D-Galf	4.39(G, H-1)	74.1	C-4 of residue I(I → G)	3.6	H-4 of residue G	3.6	H-4 of residue G
					/		3.87	H-3 of residue G	
1,6-β-D-Galp	4.38(H, H-1)	69	C-5 of residue H	/		3.66	H-4 of residue H		
		73.1	C-2 of residue H	/		/			
		77.3	C-3 of residue F(F → H)	/		/			
		80.3	C-4 of residue H	/		/			
	3.94(H, H-6)	/		/		4.44	H-1 of residue I(I → H)		
1,4-β-D-Galp	4.44(I, H-1)	77.3	C-3 of residue F(F → I)	3.56	H-2 of residue I	3.56	H-2 of residue I		
		/		3.87	H-3 of residue I	3.74	H-6 of residue H(H → I)		
		/		4.06	H-4 of residue I	/			
	4.06(I, H-4)	80.3	C-3 of residue I	3.87	H-3 of residue I	/			
		104.4	C-1 of residue F(F → I)	/		/			
T-β-D-Galp	4.44(J, H-1)	77.3	C-3 of residue F(F → J)	4.04	H-3 of residue J	3.59	H-5 of residue J		
		81.5	C-4 of residue J	/		/			
1,3-β-D-Glc	4.70(K, H-1)	76.8	C-4 of residue M(M → K)	3.46	H-2 of residue K				
		/		3.74	H-3 of residue K	/			
	3.74(K, H-3)	100.6	C-1 of residue N(N → K)	/		3.6	H-4 of residue K		
1,6-β-D-Glcp	4.44(L, H-1)	76.8	C-4 of residue M(M → L)	/		/			
	4.15(L, H-6)	102.2	C-1 of residue L(L → L)			4.66	H-1 of residue N(N → L)		
1,4-β-D-Glcp	4.52(M, H-1)	74.2	C-4 of residue O(O → M)		/	/			
	3.64(M, H-4)	103.2	C-1 of residue I(I → M)	/		/			
T-β-D-Glcp	4.66(N, H-1)	70.3	C-4 of residue N	/		3.64	H-4 of residue M(M → N)		
1,4,6-β-D-Glcp	4.66(O, H-1)	/		/		3.64	H-4 of residue M(M → O)		
	3.96(O, H-4)	101.6	C-1 of residue M(M → O)	4.15	H-5 of residue O	4.15	H-5 of residue O		
	3.91(O, H-6)	101.6	C-1 of residue M(M → O)	/		/			
T-α-D-Manp	5.25(P, H-1)	72.2	C-4 of residue S(S → P)	/		3.92	H-2 of residue P		
1,4-α-D-Xylp	5.30(Q, H-1)	70.7	C-S of residue S(S → Q)	3.54	H-2 of residue Q	3.54	H-2 of residue Q		
	4.00(Q, H-4)	99.4	C-1 of residue R(R → Q)	/		/			
T-α-D-Xylp	4.91(R, H-1)	68.9	C-5 of residue R	3.77	H-2 of residue R	3.54	H-4 of residue Q(Q → R)		
		83.5	C-4 of residue R	/		3.77	H-2 of residue R

	1,4,6-α-D-Manp	4.89(S, H-1)	69.1	C-6 of residue F(F → S)	3.45	H-2 of residue S		
		3.96(S, H-4)	99.9	C-1 of residue Q(Q → S)	/		/	
	3.66(S, H-6)	100.1	C-1 of residue P(P → S)	/		/	
ERP-A	→4)-α-GalpA-(1→	4.98(A, H-1)	77.7	C-4 of residue A (A → A)	3.7	H-2 of residue A	3.7	H-2 of residue A
					3.93	H-3 of residue A	4.32	H-4 of residue A(A → A)
					4.32	H-4 of residue A	4.31	H-4 of residue D(D → A)
		4.32(A, H-4)	70.3	C-4 of residue A	3.7	H-2 of residue A	3.93	H-2 of residue A
			71.3	C-2 of residue A	3.93	H-3 of residue A	4.98	H-1 of residue A(A → A)
			73.9	C-3 of residue A	4.98*	H-1 of residue A	5.02	H-1 of residue D(D → A)
			99.08	C-1 of residue E(E → A)			/	
			99.2	C-1 of residue D (A → D)			/	
			99.3	C-1 of residue A (A → A)			/	
	β-GalpA(1→	4.52(B, H-1)	/		3.65	H-3 of residue B	3.7	H-4 of residue D(D → B)
			/		4.32	H-2 of residue B	/	
	α-Araf-(1→	5.69(C, H-1)	70.3	C-4 of residue D(D → C)	3.67	H-3 of residue C	4	H-4 of residue C
			/		4.24	H-2 of residue C	4.24	H-2 of residue C
	→4)-α-GalpA-6-O-Me-(1→	5.02(D, H-1)	77.7	C-4 of residue A (A → D)	3.93	H-3 of residue D	3.7	H-4 of residue D
					4.31	H-4 of residue D	4.32	H-4 of residue A (A → D)
					/		4.52	H-1 of residue B
					/			
		4.31(D, H-4)	73.9	C-3 of residue D	3.73	H-2 of residue D	3.7	H-2 of residue A
			96.4	C-1 of residue B(B → D)	3.92	H-3 of residue D	3.92	H-3 of residue D
			99.08	C-1 of residue E(E → D)	/		4.98	H-1 of residue A(A → D)
			99.3	C-1 of residue A(A → D)		/	5.02	H-1 of residue E(E → D)
			107.1	C-1 of residue C(C → D)		/	/	
	→2,4)-α-Rhap-(1→	5.3(E, H-1)	/		3.92	H-4 of residue E	3.56	H-2 of residue E
			/		/		/	
		3.56(E, H-2)	71.3	C-3 of residue E	3.79	H-3 of residue E	5.02	H-1 of residue D(D → E)
			75.4	C-2 of residue E	/		/	
		3.92(E, H-4)	68.2	C-5 of residue E	/		4.32	H-3 of residue E
			/		/		4.98	H-1 of residue A(A → E)

Through the coupling signals observed between anomeric protons and carbons of various sugar residues on the HMBC long-range correlation spectrum, or between anomeric carbons and protons, and considering the robust NOESY signals often arising from the spatial proximity of protons at adjacent glycosidic linkage sites, the HMBC and NOESY spectra further facilitated the deduction of the interlinkage sequence among sugar residues. The HMBC spectrum (Figure 4D) and NOESY spectrum (Figure 4F) of ERP-N revealed coupling signals as summarized in Table 5. By combining these findings with methylation results and literature reports (16, 20, 23–26, 28–43, 46–48), a plausible repeating unit structure for ERP-N is illustrated in Figure 4I. Similarly, the plausible repeating unit structure for ERP-A is presented in Figure 5A–I.

### 3.2 Effects of AER and ERP on the body weight of rats with FD

**Figure 6:**
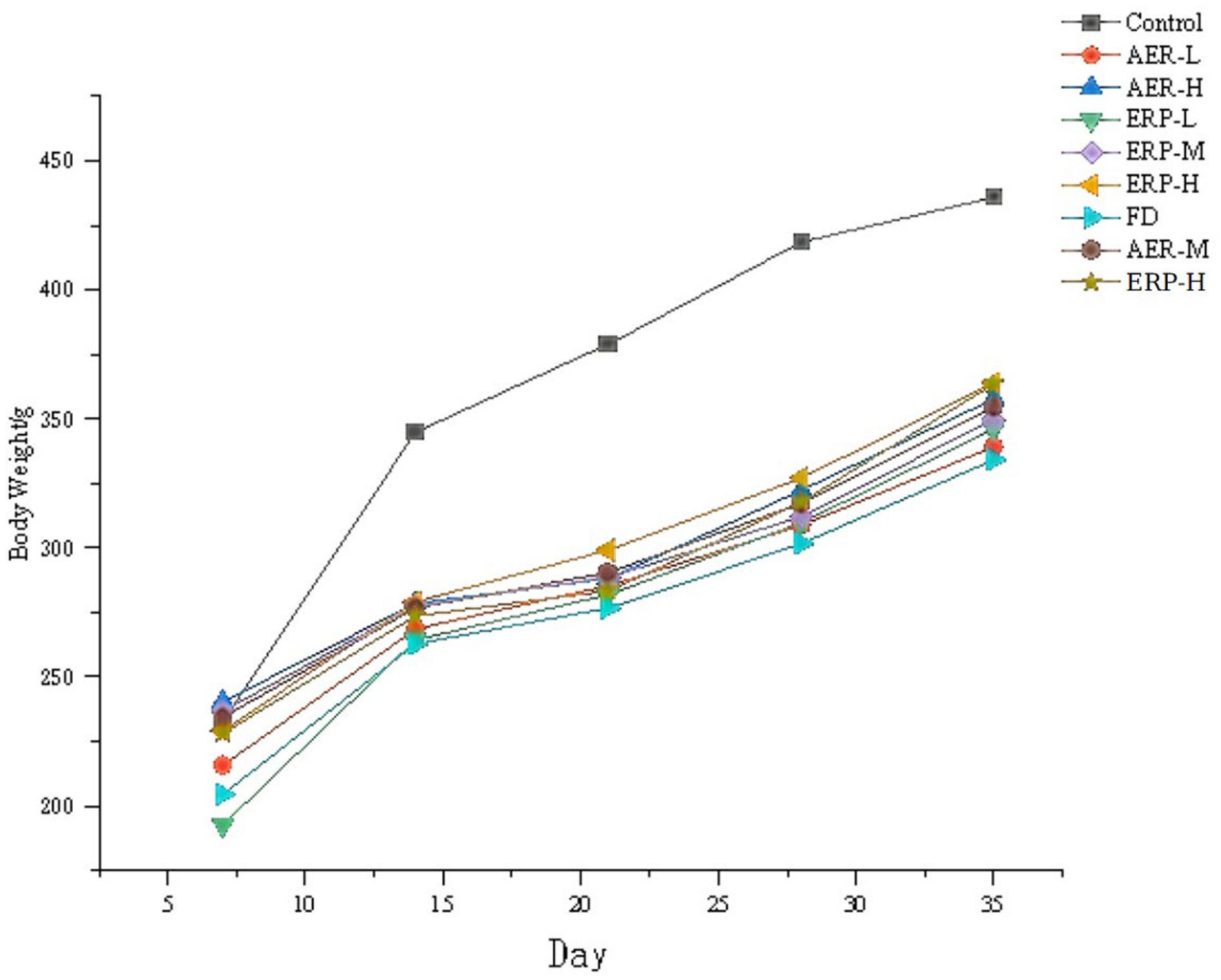
Effects of AER and ERP on the body weight of rats with FD.

Before commencing experimental modeling, an adaptive feeding regimen was implemented to ensure that all the rats demonstrated smooth coat coloration, normal behavior, and regular stool consistency (Figure 6). One week following the modeling, individual rats exhibited symptoms such as softer stool, dull coat coloration, and heightened irritability, in contrast to the control group. By the third week, these symptoms became pronounced, with significant weight differences observed when compared to the control group (Figure 6). Following a 14-days treatment regimen, the administration group demonstrated a return to normal physiological conditions, characterized by average weight gain, lustrous coat, and normal fecal output (Figure 6).

### 3.3 Histopathological examination

**Figure 7:**
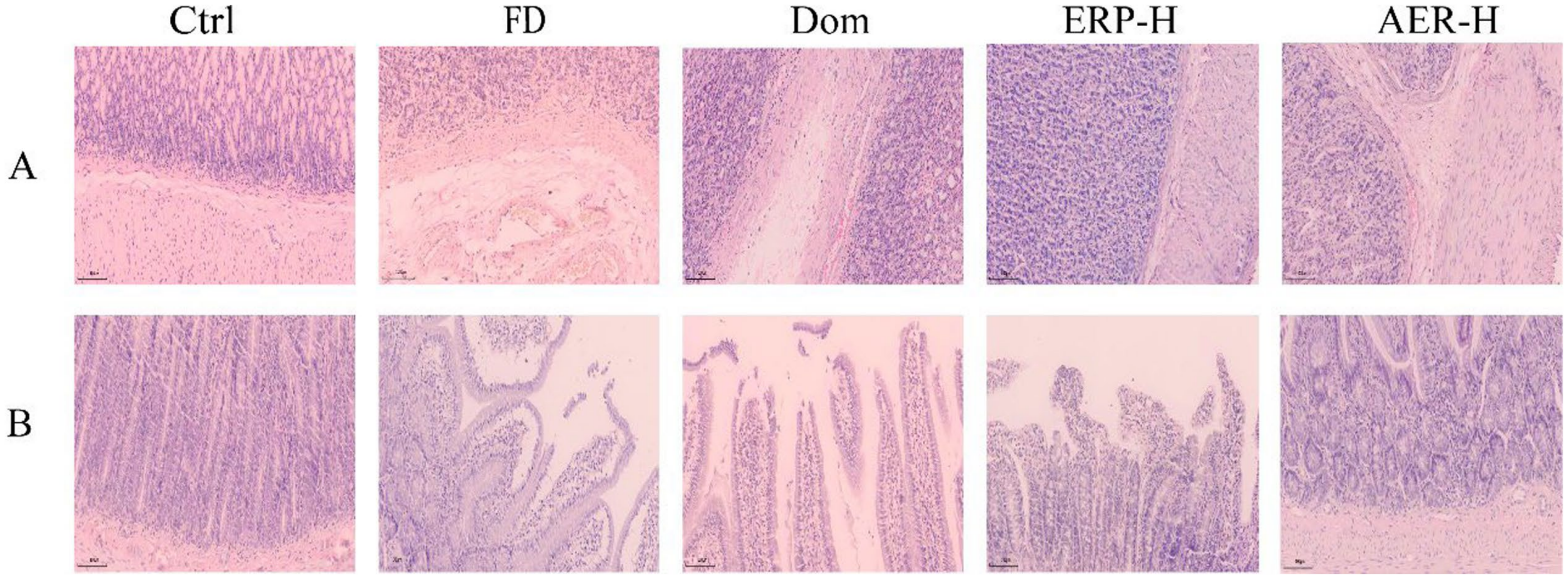
Histopathological examination of gastric antrum and duodenum tissues in rats. **(A)** H&E staining of gastric antrum, scale bar = 100 *μ*m; **(B)** H&E staining of duodenum, scale bar = 100 *μ*m.

In the control group, the gastric antrum exhibited no evidence of inflammatory cell infiltration (Figure 7A). In contrast, the model group displayed marked inflammatory cell infiltration along with epithelial cell swelling. Relative to the model group, the polysaccharide group showed a decrease in both epithelial cell swelling and the number of inflammatory cells. The duodenal tissue structure in the control group of rats was intact, with well-defined morphology and orderly arranged goblet cells (Figure 7B). Conversely, the model group demonstrated disorganized goblet cells and infiltration of eosinophilic cells. The gastrointestinal tissue of rats within this group exhibited significant inflammatory pathological alterations, distinctly differing from the control group. Importantly, the ERP-H group showed more beneficial effects in mitigating gastrointestinal inflammation, as indicated by a reduction in the number of inflammatory cells and a decrease in the extent of epithelial cell swelling (Figures 7A,B). These findings suggest that polysaccharides from *E. rugulosa* have a superior therapeutic effect compared to its aqueous extract at the same crude drug concentration in rats with FD.

### 3.4 Effects of ERP on brain-gut peptide hormones in rats with FD

**Figure 8:**
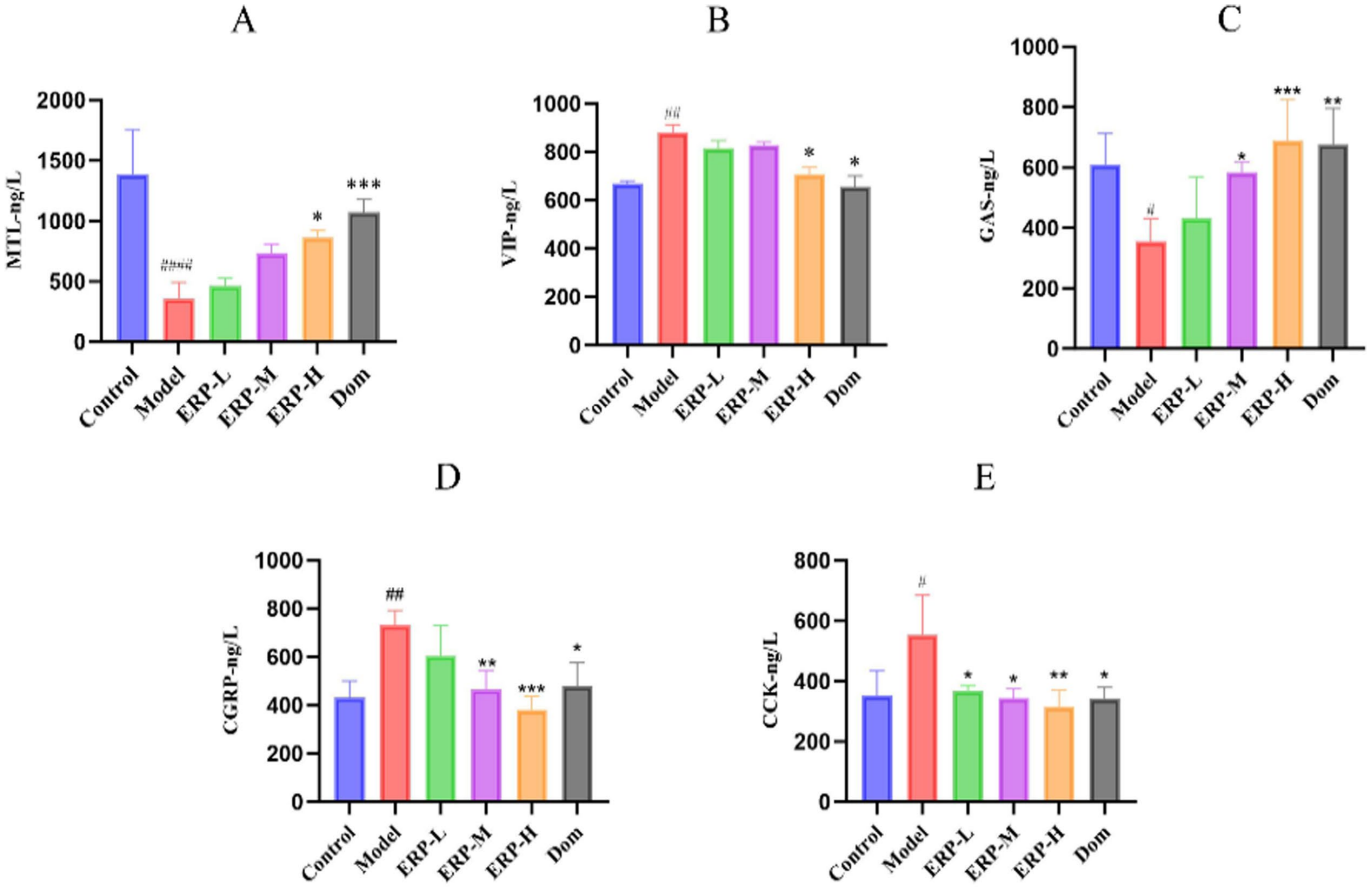
The impact of ERP on the secretion of brain-gut peptides in rats with FD. The peptides analyzed include: **(A)** Serum gastrin (GAS); **(B)** Serum motilin (MTL); **(C)** Serum cholecystokinin (CCK); **(D)** Serum vasoactive intestinal peptide (VIP); **(E)** Serum calcitonin gene-related peptide (CGRP). The data are expressed as mean ± standard deviation (SD) with a sample size of six (*n* = 6), and were subjected to one-way analysis of variance (ANOVA) for statistical evaluation. The analyses were conducted using SPSS software. Statistical significance is indicated as follows: #*p* < 0.05, ##*p* < 0.01, ###*p* < 0.001 when compared to the control group; *p < 0.05, **p < 0.01, ***p < 0.001 when compared to the model group.

Abnormal secretion of brain-gut peptides is acknowledged as a critical factor in the pathophysiology of FD. As illustrated in Figure 8, the administration of ERP at varying doses elicited distinct effects on brain-gut peptide hormone levels in rats with FD. Notably, high-dose ERP (ERP-H) resulted in a significant increase in gastrin (GAS) levels (*p* < 0.001) (Figure 8C) and also produced a marked elevation in motilin (MTL) levels (*p* < 0.05) (Figure 8A). In contrast, the administration of low-dose (ERP-L), medium-dose (ERP-M), and high-dose (ERP-H) polysaccharides resulted in a significant reduction or a trend towards a significant reduction in cholecystokinin (CCK) levels (*p* < 0.01 for all doses compared to the control) (Figure 8E). Specifically, ERP-H was associated with a notable decrease in vasoactive intestinal peptide (VIP) levels (p < 0.05) (Figure 8B). Additionally, both medium-dose (ERP-M) and high-dose (ERP-H) polysaccharides significantly lowered calcitonin gene-related peptide (CGRP) levels (Figure 8D), with ERP-H exhibiting a more substantial effect (p < 0.01 for ERP-M and p < 0.001 for ERP-H, respectively).

### 3.5 ERP treatment improved the abnormal transcription pattern associated with the pathogenesis of FD in model rats

**Figure 9:**
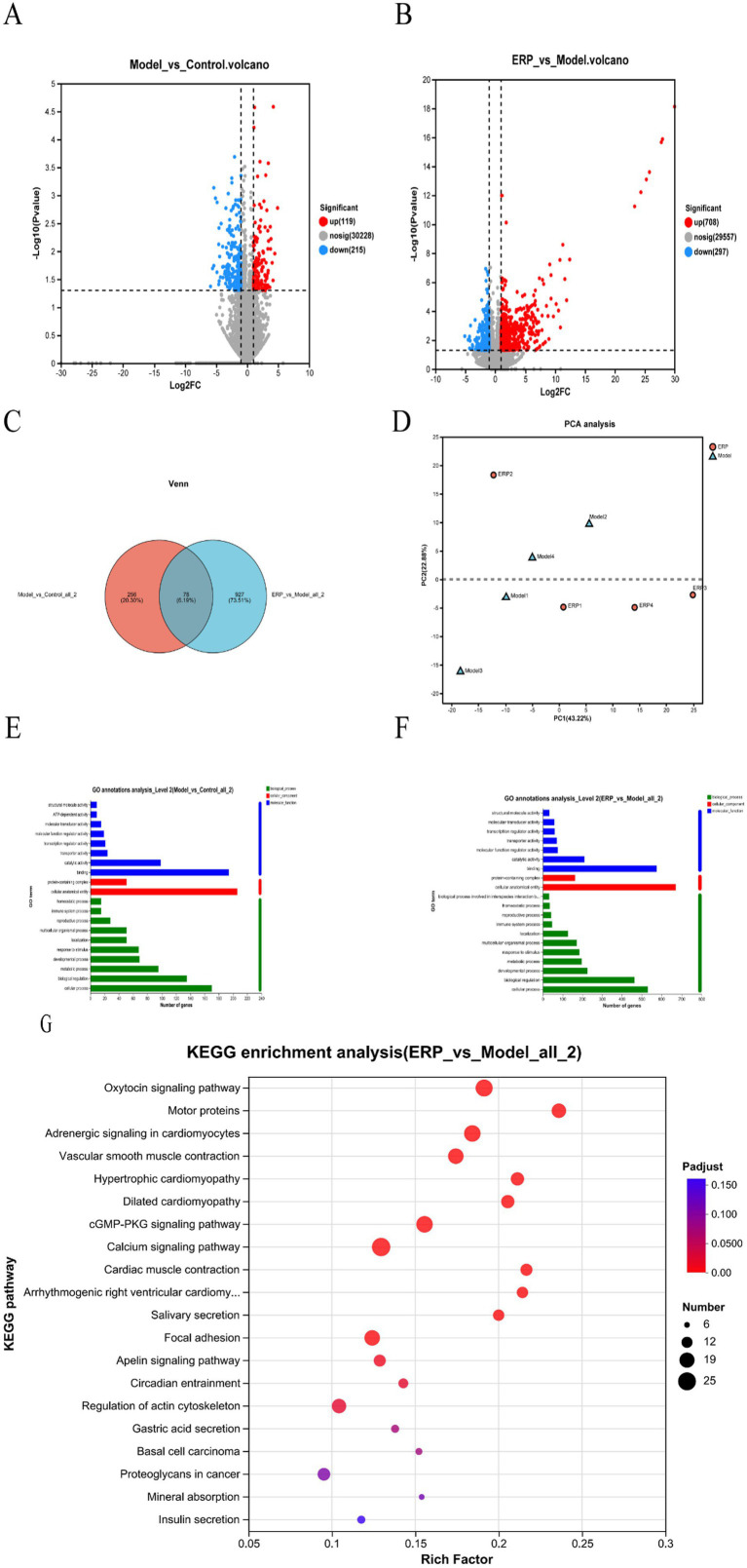
ERP treatment affected the metabolic changes associated with the pathogenesis of FD in Model rats. **(A)** Volcano plot of differentially expressed genes (DEGs) between the Model group and the Control group; **(B)** Volcano plot of DEGs between the ERP group (ERP-H) and the Model group; **(C)** Venn diagram showing the comparison of important common gene sets in transcriptome profiling between **(A,B)**; **(D)** Principal Component Analysis (PCA) was conducted to compare the ERP group (ERP-H) with the Model group; **(E)** The top 20 Gene Ontology (GO) terms were identified for genes exhibiting significant differences between the Model group and the Control group; **(F)** The top 20 GO terms were determined for genes with significant differences between the ERP group (ERP-H) and the Model group; **(G)** KEGG pathway enrichment analysis was performed to compare the ERP group (ERP-H) with the Model group.

To explore the underlying mechanisms of the therapeutic effects of ERP on gastric regulatory dysfunction in rats with FD, we performed transcriptome sequencing analysis. As illustrated in Figure 9D, there were significant differences in gene expression between the model group and the ERP-treated group. Specifically, relative to the control group (as illustrated in Figure 9A), the model group demonstrated an upregulation of 119 differentially expressed genes (DEGs) and a downregulation of 215 DEGs. In contrast, the group treated with ERP, as depicted in Figure 9B, exhibited an upregulation of 708 DEGs and a downregulation of 297 DEGs compared to the model group. Additionally, Figure 9C shows the identification of 78 DEGs common to both the Model-versus-Control and ERP-versus-Control comparisons, which may play a role in ERP’s modulation of gastric protection impairment. To further clarify these differences, a heatmap (Figure 9E) was used, which illustrates the differential gene expression between the ERP-treated and model groups, indicating elevated gene expression levels in the ERP group relative to the model group. To obtain a more comprehensive understanding of the potential prognostic mechanisms of ERP in FD rats, we performed Gene Ontology (GO) and Kyoto Encyclopedia of Genes and Genomes (KEGG) enrichment analyses. The outcomes of the Gene Ontology (GO) analysis of differentially expressed genes (DEGs) across the groups are presented in Figures 9E,F. Importantly, the Kyoto Encyclopedia of Genes and Genomes (KEGG) enrichment analysis (Figure 9G) identified the cGMP-PKG signaling pathway as a critical target. This pathway is implicated in the modulation of gastric regulatory dysfunction associated with FD through the alteration of gene expression by ERP.

### 3.6 Gut microbiota analysis

#### 3.6.1 Diversity analysis of gut microbiota

**Figure 10:**
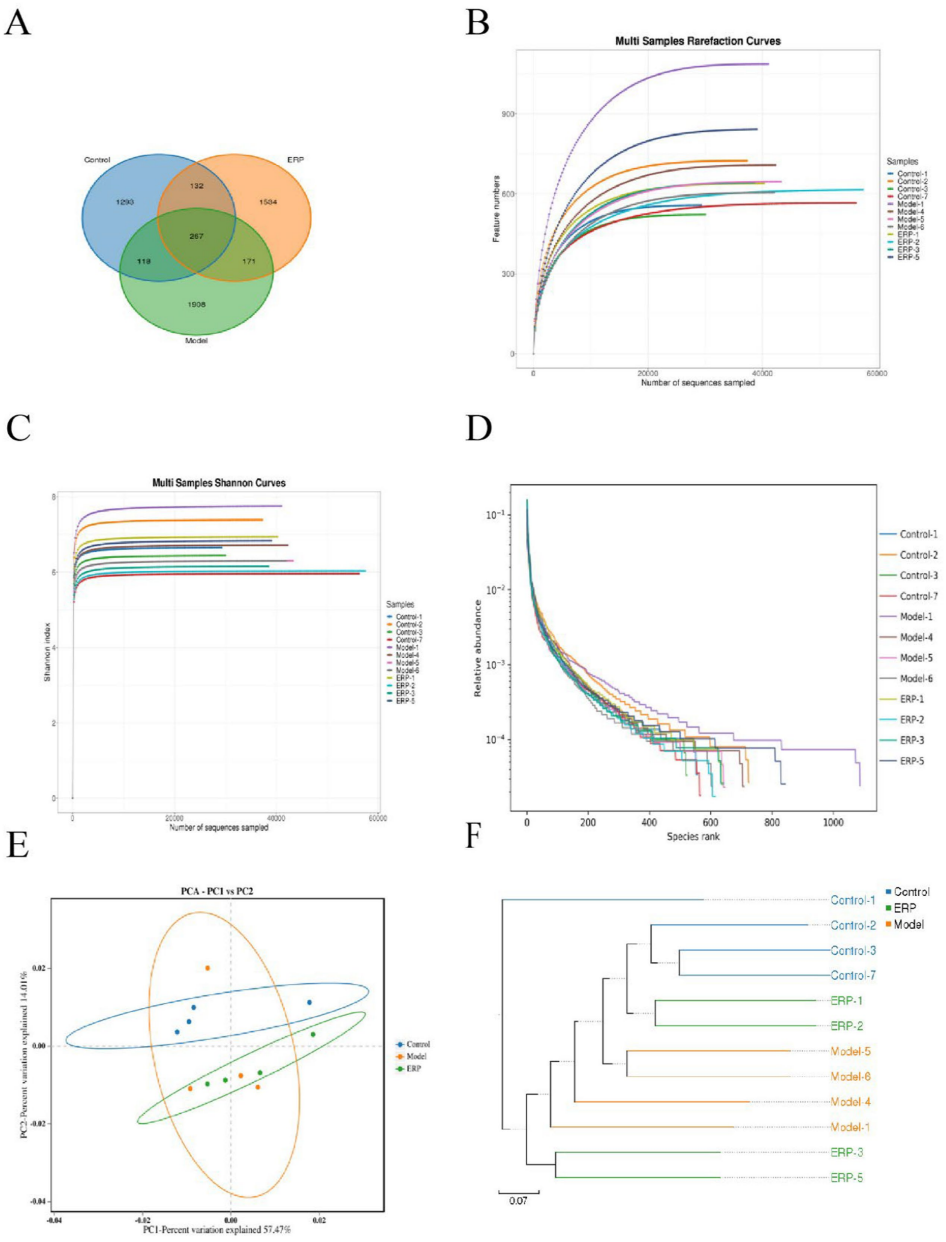
Impact of ERP on gut microbiota diversity in FD rats (*n* = 4 per group) **(A)** Venn Diagram; **(B)** Sample Rarefaction Curve; **(C)** Sample Shannon Index Curve; **(D)** Sample Rank-Abundance Curve;**(E)** PCA Analysis Plot; **(F)** Unweighted Pair Group Method with Arithmetic Mean (UPGMA) Clustering Tree of Samples. (ERP represents ERP-H).

To elucidate the impact of ERP on the richness, evenness, and diversity of the gut microbiota in rats with FD, we utilized Venn diagrams, along with Alpha and Beta diversity analyses, to evaluate the diversity indices and *α*-diversity of the samples. As illustrated in Figure 10, the Venn diagram (Figure 10A) demonstrates the number of shared and unique operational taxonomic units (OTUs) among the samples. Significantly, the number of shared Operational Taxonomic Units (OTUs) between the ERP group and the model group (171 OTUs) exceeds that between the control group and the model group (118 OTUs). The rarefaction curve (Figure 10B) corroborates the adequacy of the sample sequences for robust data analysis. Furthermore, Figure 10C illustrates a higher Shannon index, which signifies a greater diversity and richness of species, indicating that the samples have effectively captured a comprehensive representation of microbial species diversity. The rank abundance curve (Figure 10D) demonstrates that the ERP group displays greater species richness and evenness relative to the model group. Furthermore, Beta diversity analysis (Figures 10E,F) was performed to evaluate the similarity in species diversity across various samples. The findings indicate that the ERP-H group exhibits a closer resemblance to the control group than to the model group, implying that ERP-H may have the capacity to restore gut microbiota in rats with FD.

#### 3.6.2 ERP increases the abundance of beneficial gut microbiota and reduces the abundance of pathogenic bacteria

**Figure 11:**
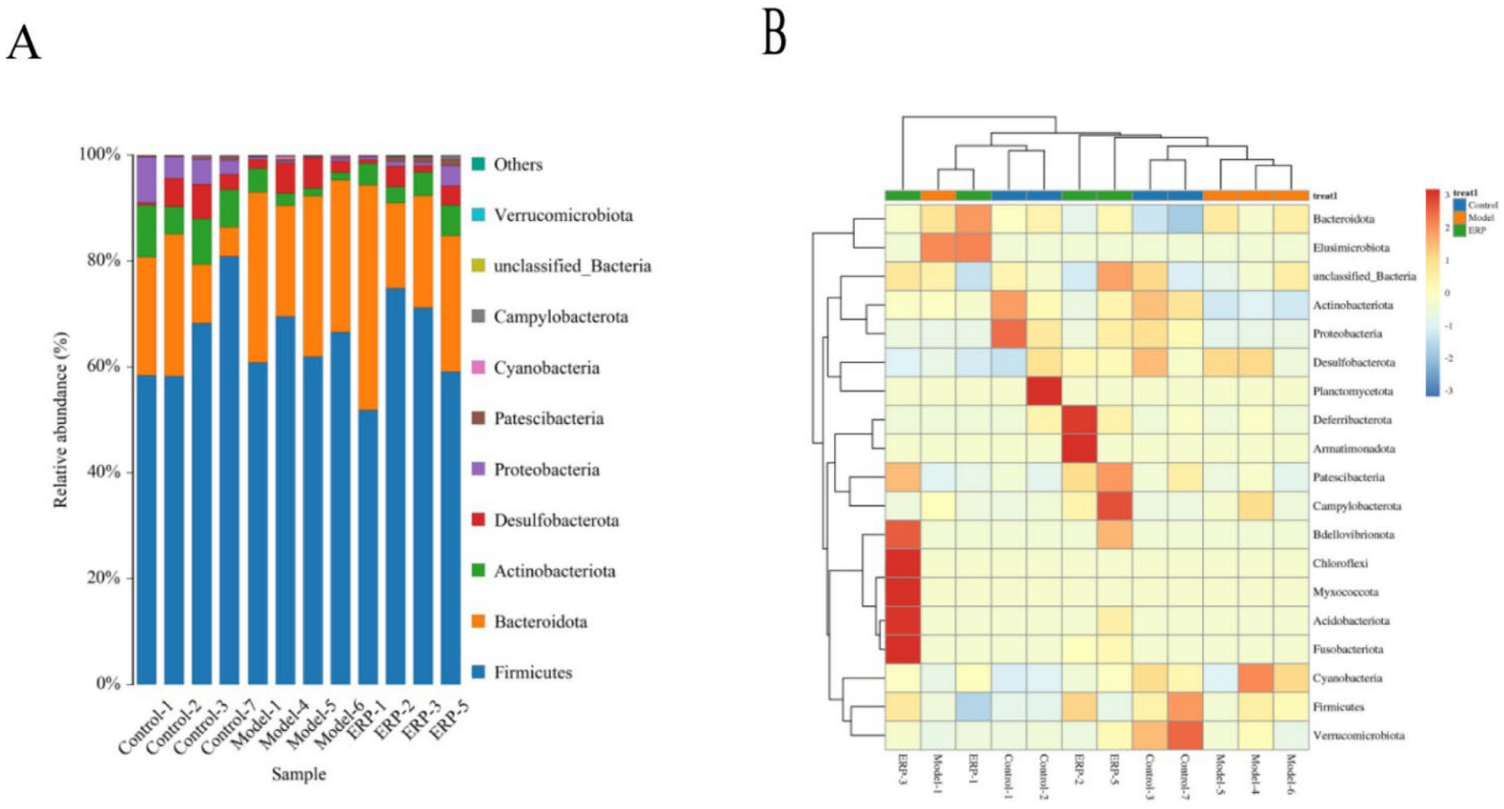
ERP increases the abundance of beneficial gut microbiota and reduces the abundance of pathogenic bacteria. **(A)** Species distribution at the phylum level, with sample names on the x-axis and relative abundance percentages on the y-axis. Different colors represent different species, and stacked bars indicate the top 10 taxa in terms of relative abundance at each taxonomic level; **(B)** Clustering heatmap of species abundance at the phylum level, with samples and groups represented horizontally (the bar plot above shows the corresponding group information), and species taxa represented vertically. The clustering tree on the left is for species, and the clustering tree above is for samples, reflecting the similarity in community composition among samples. The values corresponding to the heatmap are Z-scores obtained after standardizing the relative abundance of each species in each row. The heatmap color gradient ranges from blue to red, indicating an increase in the relative proportion of a species across different samples. Comparisons can only be made between samples, not between species.

The gut microbiota significantly influences the emergence and progression of FD through its effects on gastrointestinal motility. One of the primary pathological mechanisms associated with FD is abnormal gastrointestinal motility, characterized by delayed gastric emptying and impaired gastric accommodation. Dysbiosis within the gut microbiota may disrupt gastrointestinal motility by modulating the function of the intestinal neuro-muscular system, thereby contributing to the onset and progression of FD. As demonstrated in Figures 11A,B, the analysis of the species’ evolutionary tree and the genus-level species distribution map reveals a marked decrease in the abundance of the harmful bacterial genus Bacteroidetes following ERP (ERP-H) treatment. In contrast, Firmicutes, which play a vital role in energy metabolism and immune regulation within the gut, exhibited a significant increase in their relative abundance after ERP (ERP-H) treatment. Proteobacteria, which serve multiple functions in the gut microbiota, including maintaining ecological balance and diversity, as well as participating in metabolism and nutrient processing, also showed notable changes. In particular, the control group exhibited the highest relative abundance of Proteobacteria, followed by the ERP group (ERP-H), whereas the model group demonstrated the lowest abundance. Patescibacteria, another significant constituent of the gut microbiota, may play a pivotal role in sustaining intestinal ecological balance, participating in metabolic processes, regulating immune responses, and being associated with various disease states. Importantly, the ERP (ERP-H) treatment resulted in a significant increase in the relative abundance of this bacterial group.

### 3.7 Correlation analysis between beneficial and key gut microbiota and the transcriptome

**Figure 12:**
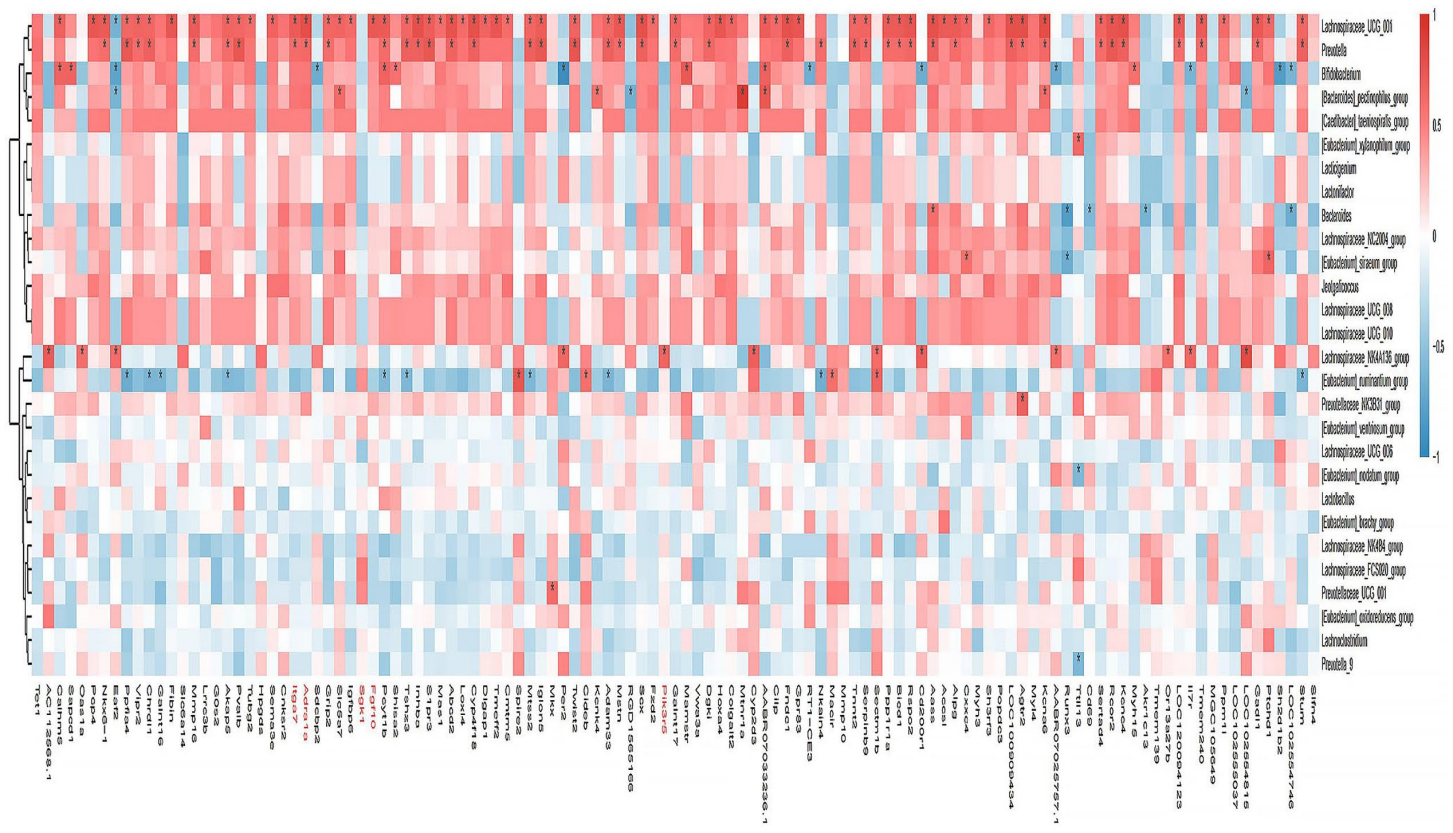
Correlation analysis between beneficial gut microbiota and the transcriptome is shown in a heatmap. It illustrates the relationship between the common gene set and beneficial bacteria using 16S rRNA data. Red signifies a positive correlation, and blue signifies a negative one. The red gene set is linked to the PI3K/Akt/cGMP-PKG signaling pathway.

Prior research has shown significant progress in the investigation of diverse diseases through integrated analysis of transcriptomic data and 16S rRNA gut microbiota profiles. Notably, within the domains of intestinal inflammatory diseases, metabolic disorders, and neurodegenerative conditions, these combined analyses have revealed complex associations between host gene expression patterns and the composition of gut microbiota. These findings provide new insights into the underlying pathogenesis and potential therapeutic strategies for these diseases. As illustrated in Figure 12, significant correlations are observed between specific gut microbiota members and transcriptome gene sets. Notably, pathogenic bacteria, such as those from the Bacteroides genus, demonstrate a negative correlation with certain transcriptome gene sets. In contrast, beneficial bacteria, including members of the Lachnospiraceae family and the Prevotella genus, show a positive correlation with other transcriptome gene sets. These observations indicate a reciprocal relationship between modifications in gene expression and alterations in gut microbiota composition, implying potential mechanisms through which the gut microbiota may affect host physiology and disease progression. Moreover, it is important to acknowledge that the interaction between the transcriptome and gut microbiota is likely bidirectional. Alterations in host gene expression have the potential to affect the composition and functionality of gut microbiota. Conversely, modifications in gut microbiota can reciprocally influence host gene expression through diverse mechanisms, including the production of metabolites, modulation of the immune system, and direct interactions with host cells. In summary, the integrated analysis of transcriptome and gut microbiota serves as a robust approach for elucidating the intricate interactions between the host and its microbial residents. This methodology offers novel insights into disease pathogenesis and facilitates the development of targeted therapeutic strategies.

### 3.8 Activation of the PI3K/Akt/cGMP-PKG signaling pathway by ERP ameliorates functional dyspepsia (FD)

**Figure 13:**
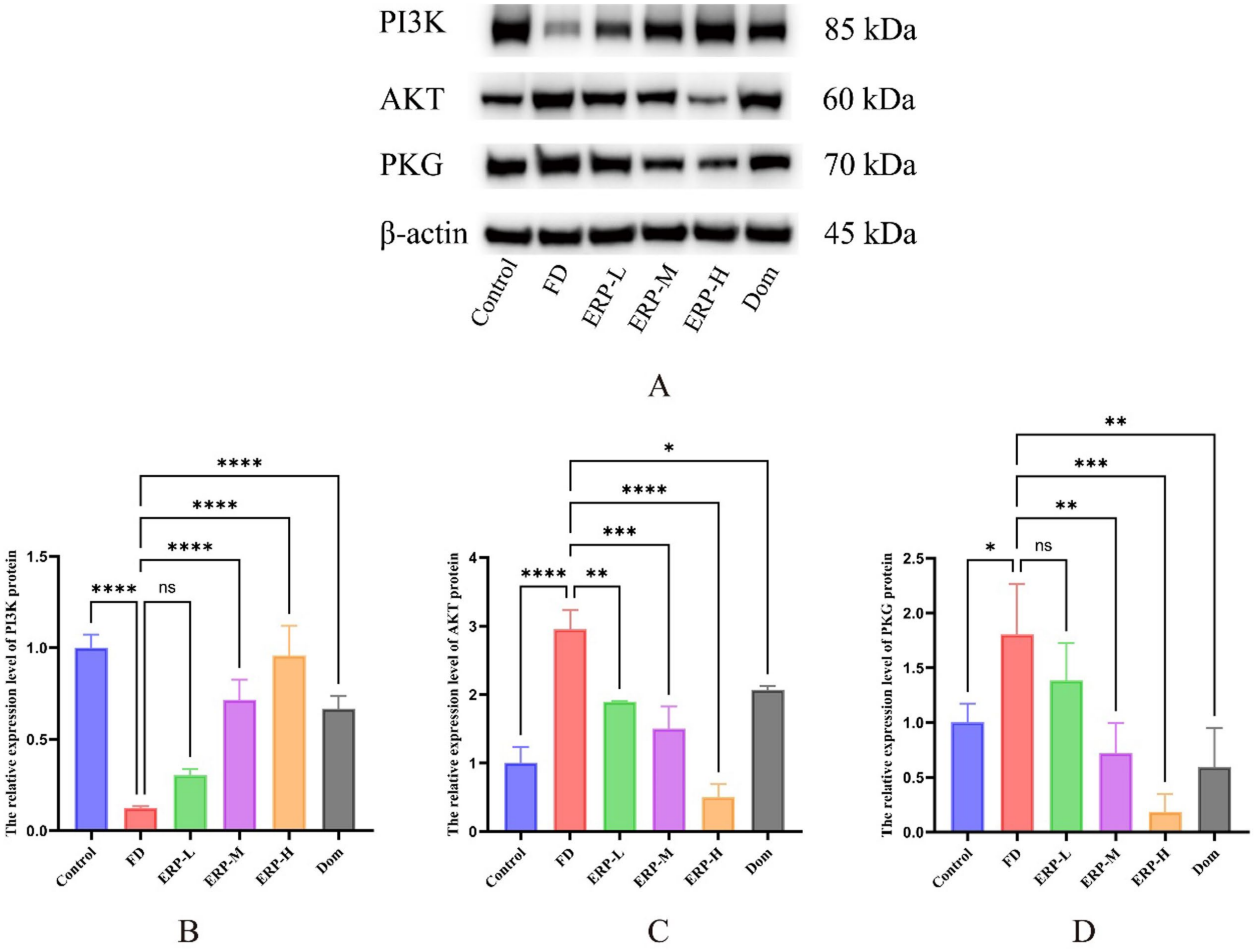
Western blotting was used to detect the relative levels of proteins involved in the PI3K/AKT/cGMP-PKG signaling pathway in model rats before and after FD treatment. **(A)** Effect of ERP on the expression of PI3K, AKT, and PKG proteins. *β*-actin was used as the loading control. **(B)** Expression level of PI3K protein. **(C)** Expression level of AKT protein. **(D)** Expression level of PKG protein. The data are presented as the mean ± SD (*n* = 3). ****p < 0.0001, ***p < 0.001, **p < 0.01, *p < 0.05 compared with the Model group.

To investigate the role of the PI3K/Akt/cGMP-PKG signaling pathway in the management of functional dyspepsia (FD), we conducted an analysis of the expression levels of key proteins involved. As shown in Figures 13A–C, ERP treatment led to an increase in PI3K protein expression and a decrease in AKT protein expression in the stomach of rats, compared to the model group. PKGs serve as pivotal effectors in the cGMP-PKG signaling cascade, where their phosphorylation triggers a series of downstream events, encompassing smooth muscle relaxation, inhibition of apoptosis, and modulation of tissue permeability (49). Furthermore, as depicted in Figure 13D, ERP treatment resulted in a decreased expression of PKG protein within the stomach tissue of rats with functional dyspepsia, when compared to the model group.

### 3.9 Evaluation of *in vitro* immunomodulatory effects of purified polysaccharides from *E. rugulosa*

#### 3.9.1 Effects of ERP-N and ERP-A on the viability of RAW264.7 cells

**Figure 14:**
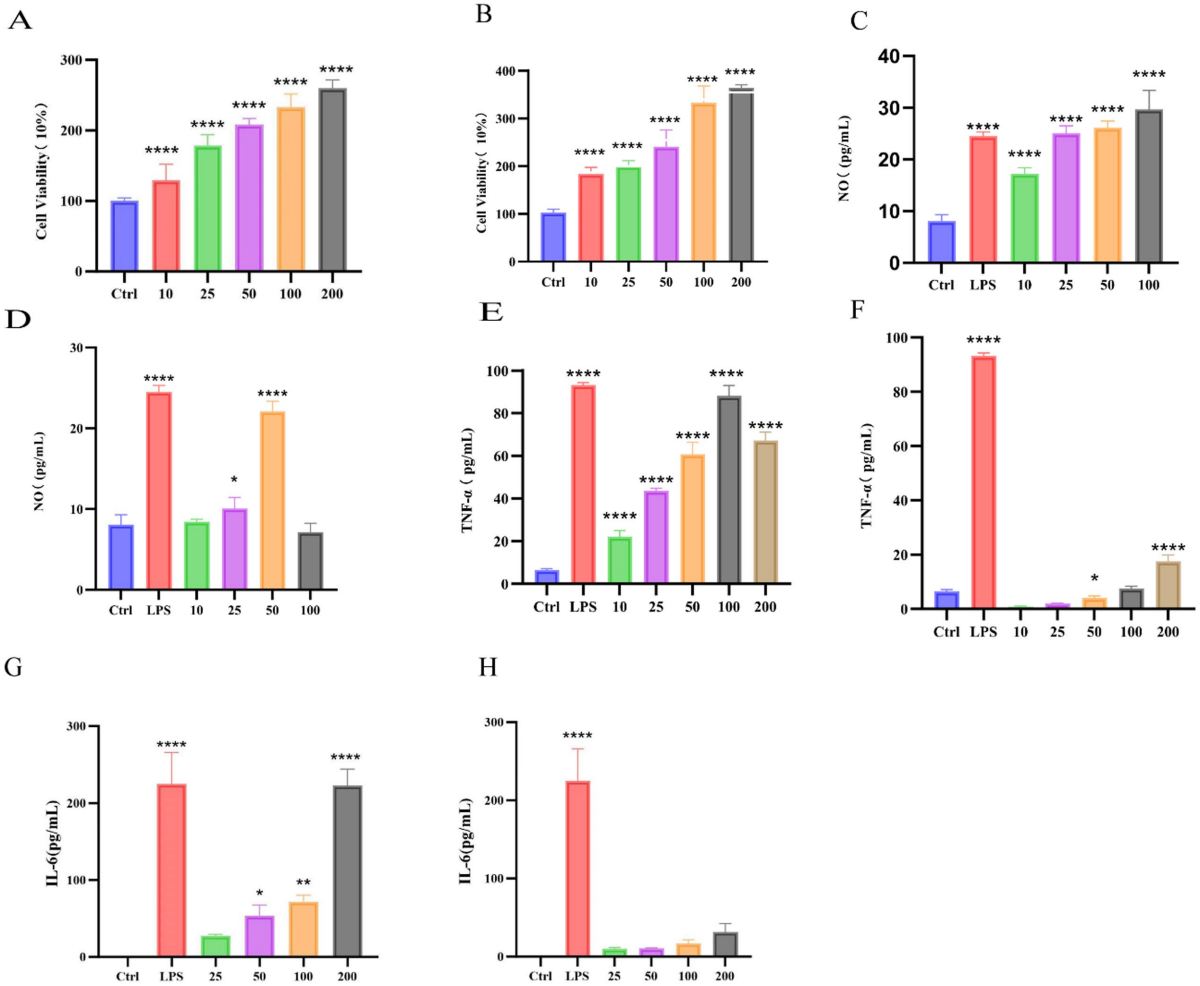
*In vitro* studies were conducted to evaluate the effects of ERP-N and ERP-A on various biological parameters in RAW 264.7 cells. **(A)** the impact of ERP-N on cell viability; **(B)** the influence of ERP-A on cell viability; **(C)** the effect of ERP-N on NO production; **(D)** the effect of ERP-A on NO production; **(E)** the influence of ERP-N on TNF-*α* production; **(F)** the influence of ERP-A on TNF-*α* production; **(G)** the effect of ERP-N on IL-6 production; **(H)** the effect of ERP-A on IL-6 production in RAW 264.7 cells. Statistical significance is denoted as follows: *p < 0.05, **p < 0.01, ***p < 0.001, and ****p < 0.0001, in comparison to the control group.

In this study, our objective was to evaluate the immunomodulatory effects of purified polysaccharides derived from *E. rugulosa* on RAW264.7 macrophages. We commenced by employing the CCK8 assay to examine the effects of ERP-N and ERP-A on the viability of these cells. As illustrated in Figures 14A,B, both ERP-N and ERP-A did not demonstrate any inhibitory effects on cell viability across the concentration range of 10–200 μg/mL, relative to the control group. Consequently, informed by these findings, we selected concentrations within the range of 10–200 μg/mL for subsequent experimental procedures.

#### 3.9.2 Effects of ERP-N and ERP-A on nitric oxide (NO) production

Nitric oxide (NO) secretion, a critical mediator in immune responses, is utilized as a marker for macrophage activation. As depicted in Figures 14C,D, both lipopolysaccharide (LPS) and ERP-N, at concentrations ranging from 10 to 100 μg/mL, markedly elevated NO production in RAW264.7 cells (*p* < 0.001). Conversely, ERP-A significantly augmented NO production in these cells only at the concentrations of 25 and 50 μg/mL.

#### 3.9.3 Effects of ERP-N and ERP-A on cytokine secretion

To further investigate the potential immunomodulatory properties of ERP-N and ERP-A, we assessed their impact on the secretion of cytokine TNF-*α*. The ERP-N treatment, at concentrations ranging from 10 to 200 μg/mL, significantly enhanced the secretion of TNF-*α* and IL-6 in a dose-dependent manner Figures 14E,F. Conversely, ERP-A exhibited either negligible changes (Figures 14G,H) or the effects were limited to specific concentrations (50 and 200 μg/mL), which did not demonstrate a clear dose-dependent relationship.

## 4 Discussion

In this study, we examined the pharmacological effects of a polysaccharide, designated as ERP, which was extracted and purified from *E. rugulosa*, on the treatment of functional dyspepsia (FD). Additionally, preliminary investigations were undertaken to elucidate the structural characteristics and immunomodulatory activities of the homogeneous polysaccharides, ERP-N and ERP-A, isolated from ERP. Our results indicate that ERP significantly mitigates disturbances in intestinal flora and gastric regulatory disorders in the FD model rats. This therapeutic effect may be associated with gut microbiota remodeling and modulation of the PI3K/Akt/cGMP-PKG-related pathway, although a direct causal relationship remains to be further confirmed.

FD is a common chronic upper gastrointestinal disease that has a passive effect on the health of patients. In terms of mechanism of action, TCM can prevent and treat FD by regulating brain-gut peptides, regulating gastrointestinal hormone content, improving gastric motility, regulating immune function, regulating intestinal microbiota, and regulating inflammatory response (50). Brain-gut peptides, which are neuroactive polypeptides secreted by both the central nervous system and the gastrointestinal tract, exhibit diverse functions in gastrointestinal physiology (7). These peptides are involved in the regulation of gastric acid secretion, gastrointestinal motility, vasodilation, and the modulation of anti-inflammatory responses (50). Abnormal secretion of these peptides significantly affects gastrointestinal function, resulting in dysfunction. Acting as a critical link between the central nervous system and the gastrointestinal tract, brain-gut peptides play a vital role in maintaining normal digestive physiology by regulating gastrointestinal motility, secretion, and sensory functions (50). Our experimental results indicate that ERP-H administration leads to an increase in motilin (MTL) and gastrin (GAS) levels in rat serum, while concurrently reducing the concentrations of cholecystokinin (CCK), vasoactive intestinal peptide (VIP), and calcitonin gene-related peptide (CGRP), in comparison to the model group. Based on these findings, we propose that ERP-H may mitigate gastric regulatory dysfunction induced by FD, thereby modulating gastrointestinal function. To further elucidate this hypothesis, we conducted a transcriptome analysis to investigate aberrant transcriptional patterns linked to the pathogenesis of FD in FD-model rats treated with ERP-H.

Prior research has implicated the PI3K/Akt/cGMP-PKG signaling pathway in FD and disturbances of the intestinal microbiota, suggesting that these factors may affect the pathway’s functionality (51). Probiotics, including strains such as Lactobacillus and Bifidobacterium, may exert an indirect influence on the PI3K/Akt/cGMP-PKG pathway by modulating immune responses, enhancing nutrient absorption, and inhibiting the proliferation of pathogenic bacteria (52, 53). Opportunistic pathogens, including specific strains of Enterobacteriaceae, can modulate the PI3K/Akt/cGMP-PKG signaling pathway under particular conditions by generating inflammatory mediators and metabolites (54–56). Concurrently, short-chain fatty acid-producing bacteria such as Bacteroides and Prevotella influence the function of intestinal mucosal barrier, immune responses, and inflammatory processes through the production of acetic acid, propionic acid, and butyric acid (57). These activities, in turn, indirectly impact the PI3K/Akt/cGMP-PKG pathway (58). Our experimental findings reveal a positive correlation between beneficial bacterial taxa, such as Lachnospiraceae (59), and the expression of genes associated with the PI3K/Akt/cGMP-PKG pathway, whereas detrimental bacterial taxa, including specific Bacteroides species (14), exhibit a negative correlation. This study provides evidence that ERP-H may alleviate the symptoms of functional dyspepsia (FD) by promoting the proliferation of beneficial bacteria while inhibiting the growth of pathogenic bacteria, potentially through the modulation of the PI3K/Akt/cGMP-PKG signaling pathway.

Polysaccharides, a complex subclass of carbohydrates, are typically not directly digested and absorbed by the human body. Instead, they undergo metabolic degradation facilitated by a spectrum of polysaccharide-degrading enzymes secreted by gut microbiota (14). These enzymes, encompassing polysaccharide lyases, glycoside hydrolases, and carbohydrate esterases, catalyze the breakdown of polysaccharides into low-molecular-weight oligosaccharides or monosaccharides, which are readily absorbed and utilized by the human body. From the polysaccharides isolated from *E. rugulosa*, we have successfully purified two homogeneous polysaccharides: a neutral polysaccharide termed ERP-N, with a molecular weight of 13,415 Da, and an acidic polysaccharide designated ERP-A, with a molecular weight of 3,691 Da. The monosaccharide composition of ERP-A includes mannose, rhamnose, glucose, galactose, xylose, and arabinose. In contrast, ERP-N comprises mannose, rhamnose, glucose, galactose, xylose, and arabinose. The primary linkage types identified in ERP-N are 6-linked galactose phosphate (6-Gal(p)), 4-linked galactose phosphate (4-Gal(p)), and 4-linked glucose phosphate (4-Glc(p)), whereas in ERP-A, they are 4-linked galacturonic acid phosphate (4-GalA(p)) and terminal galacturonic acid phosphate (t-GalA(p)). The NMR spectroscopy further substantiates the probable linkage patterns between the main chain and side chains of these polysaccharides.

However, this study has several limitations. Although integrated 16S rRNA sequencing and transcriptomic analysis revealed associations between ERP-induced gut microbiota changes and host signaling pathways, the present data remain correlative. Fecal microbiota transplantation, antibiotic-mediated microbiota depletion, or pseudo-germ-free animal models will be necessary in future studies to determine whether gut microbiota changes directly mediate the therapeutic effects of ERP on FD.

## 5 Conclusion

In conclusion, polysaccharides derived from cauliflower with a tomentose surface exhibit diverse therapeutic potential in the treatment of FD in rat models. These polysaccharides have been shown to enhance body weight, mitigate pathological alterations in the gastric antrum and duodenum, and correct imbalances in brain-gut peptide regulation. The pharmacological mechanisms underlying these effects may involve modulation of the intestinal microbiota composition and activation of the PI3K/Akt/cGMP-PKG signaling pathway, thereby facilitating the relaxation of smooth gastric muscle. The weight-average molecular weights (MW) of ERP-N and ERP-A were determined as 13,415 Da and 3,691 Da, respectively. A comprehensive suite of analytical techniques, encompassing UV, IR, GC–MS, and NMR spectroscopy, was employed to elucidate the chemical compositions of these polymers. The results indicated that ERP-N consists primarily of Gal and Glc, with its side chains individually comprising Ara, Xyl, and Man. In contrast, the main structure of ERP-A is constructed from Gal, GalA, and Rha. Notably, the neutral polysaccharide ERP-N isolated from *E. rugulosa* demonstrates potential immunoregulatory activities. Elucidating the active constituents and mechanisms of action of *E. rugulosa* in the treatment of FD not only fortifies the scientific rationale behind its traditional and folk medicinal uses but also fosters the development of this herbal medicine within the realm of ethnic medicine. Further research in this area may lead to the identification of novel therapeutics.

## Data Availability

The raw data supporting the conclusions of this article will be made available by the authors, without undue reservation.

## References

[1] Camilleri M Bueno L de Ponti F Fioramonti J Lydiard RB Tack J. Pharmacological and pharmacokinetic aspects of functional gastrointestinal disorders. Gastroenterology. (2006) 130:1421–34. doi: 10.1053/j.gastro.2005.08.062, 16678556

[2] He Q. Liu C. Wang T. Sun J. Zhou X. Wang Y. . Research on mechanism of the effect of charred hawthorn on digestive by SCF/c-kit pathway. (2020). doi:10.21203/rs.3.rs-138593/v1

[3] Ford AC Mahadeva S Carbone MF Lacy BE Talley NJ. Functional dyspepsia. Lancet. (2020) 396:1689–702. doi: 10.1016/S0140-6736(20)30469-4, 33049222

[4] Liu B Kou Z Chen B. Effects and mechanisms of traditional Chinese medicines on functional dyspepsia: a review. Chin Herb Med. (2023) 15:516–25. doi: 10.1016/j.chmed.2023.06.001, 38094020 PMC10715891

[5] Kwon CY Ko SJ Lee B Cha JM Yoon JY Park JW. Acupuncture as an add-on treatment for functional dyspepsia: a systematic review and Meta-analysis. Front Med (Lausanne). (2021) 8:682783. doi: 10.3389/fmed.2021.682783, 34381798 PMC8350114

[6] Liu Y Liao W Liu X Hu Y Zhu X Ju L . Digestive promoting effect and mechanism of jiao sanxian in rats. J Ethnopharmacol. (2021a) 278:114334. doi: 10.1016/j.jep.2021.114334, 34126213

[7] Tu Y Luo X Liu D Li H Xia H Ma C . Extracts of Poria cocos improve functional dyspepsia via regulating brain-gut peptides, immunity and repairing of gastrointestinal mucosa. Phytomedicine. (2022) 95:153875. doi: 10.1016/j.phymed.2021.153875, 34911003

[8] Enck P Azpiroz F Boeckxstaens G Elsenbruch S Feinle-Bisset C Holtmann G . Functional dyspepsia. Nat Rev Dis Primers. (2017) 3:17081. doi: 10.1038/nrdp.2017.81 29099093

[9] Xiao Z Xu J Tan J Zhang S Wang N Wang R . Zhizhu Kuanzhong, a traditional Chinese medicine, alleviates gastric hypersensitivity and motor dysfunction on a rat model of functional dyspepsia. Front Pharmacol. (2022) 13:1026660. doi: 10.3389/fphar.2022.1026660, 36467071 PMC9712737

[10] Wang Y Lv M Wang T Sun J Wang Y Xia M . Research on mechanism of charred hawthorn on digestive through modulating "brain-gut" axis and gut flora. J Ethnopharmacol. (2019) 245:112166. doi: 10.1016/j.jep.2019.112166, 31421184

[11] Yang F Pu HY Yaseen A Chen B Li F Gu YC . Terpenoid and phenolic derivatives from the aerial parts of Elsholtzia rugulosa and their anti-inflammatory activity. Phytochemistry. (2021) 181:112543. doi: 10.1016/j.phytochem.2020.112543, 33161176

[12] Zheng C Yang S Huang D Mao D Chen J Zhang C . GC-MS combined with multivariate analysis for the determination of the geographical origin of Elsholtzia rugulosa Hemsl Yunnan Province. RSC Adv. (2022) 12:21287–96. doi: 10.1039/D2RA02876J, 35975083 PMC9345011

[13] Liu L Gao Q Zhang Z Zhang X. Elsholtzia rugulosa: phytochemical profile and antioxidant, anti-Alzheimer's disease, antidiabetic, antibacterial, cytotoxic and hepatoprotective activities. Plant Foods Hum Nutr. (2022) 77:62–7. doi: 10.1007/s11130-021-00941-4, 34853948

[14] Li P Xiao N Zeng L Xiao J Huang J Xu Y . Structural characteristics of a mannoglucan isolated from Chinese yam and its treatment effects against gut microbiota dysbiosis and DSS-induced colitis in mice. Carbohydr Polym. (2020) 250:116958. doi: 10.1016/j.carbpol.2020.116958, 33049862

[15] Funami T Nakauma M. Cation-responsive food polysaccharides and their usage in food and pharmaceutical products for improved quality of life. Food Hydrocoll. (2023) 141:108675. doi: 10.1016/j.foodhyd.2023.108675

[16] Wang M Liu Y Qiang M Wang J. Structural elucidation of a pectin-type polysaccharide from *Hovenia dulcis* peduncles and its proliferative activity on RAW264.7 cells. Int J Biol Macromol. (2017) 104:1246–53. doi: 10.1016/j.ijbiomac.2017.07.004, 28715863

[17] Liu Y Ye Y Hu X Wang J. Structural characterization and anti-inflammatory activity of a polysaccharide from the lignified okra. Carbohydr Polym. (2021b) 265:118081. doi: 10.1016/j.carbpol.2021.118081, 33966845

[18] Gao X Qi J Ho CT Li B Mu J Zhang Y . Structural characterization and immunomodulatory activity of a water-soluble polysaccharide from Ganoderma leucocontextum fruiting bodies. Carbohydr Polym. (2020) 249:116874. doi: 10.1016/j.carbpol.2020.116874, 32933694

[19] Ji E Wang T Guo F Zhang Y Tang C Tang D . Xiaoerfupi alleviates the symptoms of functional dyspepsia by regulating the HTR3A and c-FOS. Biomed Pharmacother. (2019) 120:109442. doi: 10.1016/j.biopha.2019.109442, 31546083

[20] Zhang S Lin L Liu W Zou B Cai Y Liu D . Shen-Ling-Bai-Zhu-San alleviates functional dyspepsia in rats and modulates the composition of the gut microbiota. Nutr Res. (2019) 71:89–99. doi: 10.1016/j.nutres.2019.10.001, 31757632

[21] Xiao HL Xiao YJ Wang Q Chen ML Jiang AL. Moxibustion regulates gastrointestinal motility via HCN1 in functional dyspepsia rats. Med Sci Monit. (2021) 27:e932885. doi: 10.12659/MSM.932885, 34845181 PMC8642983

[22] Ye Y Wang XR Zheng Y Yang JW Yang NN Shi GX . Choosing an animal model for the study of functional dyspepsia. Can J Gastroenterol Hepatol. (2018) 2018:1–13. doi: 10.1155/2018/1531958, 29623262 PMC5830275

[23] Li Y Ren M Yan H Luo L Fang X He L . Purification, structural characterization, and immunomodulatory activity of two polysaccharides from *Portulaca oleracea* L. Int J Biol Macromol. (2024) 264:130508. doi: 10.1016/j.ijbiomac.2024.130508, 38428780

[24] Shaoping N Danfei H Junyi Y Mingyong X. The Progress on characterization and functional activity of polysaccharide in food. Chin J Food Sci. (2011) 11:46–57. doi: 10.16429/j.1009-7848.2011.09.022

[25] Yuhong L Fengshan W. Applications of nuclear magnetic resonance spectroscopy in structural analysis of polysaccharides. J. Food Drug. (2007) 8:39–43. doi: 10.1021/JF505172m

[26] Hui Z Shaoping N Lianzhong A Yongjun X Zhiqiang X Guangqiang W. Review on structure and characterization methodology of polysaccharides from Ganoderma. Chin J Food Sci. (2020) 20:290–301. doi: 10.16429/j.1009-7848.2020.01.038

[27] Ming L Chunxia L Xianliang X. Application of nuclear magnetic resonance Technology in the Study of chemical structures of carbohydrate compounds. Chin. J. Pharmacol. (2009) 44:324–7. doi: 10.1016/J.JEP.2021.114334

[28] Bai H-X Gao Y-X Wang S Ma G-Y Zhao W Li X-Q . Structure characteristics of a novel pectic polysaccharide from fructus Corni and its protective effect on alcoholic fatty liver. Carbohydr Polym. (2025) 352:123153. doi: 10.1016/j.carbpol.2024.123153, 39843058

[29] Dourado F Cardoso S Silva A Gama F Coimbra M. NMR structural elucidation of the arabinan from *Prunus dulcis* immunobiological active pectic polysaccharides. Carbohydr Polym. (2006) 66:27–33. doi: 10.1016/j.carbpol.2006.02.020

[30] Fu YP Peng X Zhang CW Jiang QX Li CY Paulsen BS . *Salvia miltiorrhiza* polysaccharide and its related metabolite 5-methoxyindole-3-carboxaldehyde ameliorate experimental colitis by regulating Nrf2/Keap1 signaling pathway. Carbohydr Polym. (2023) 306:120626. doi: 10.1016/j.carbpol.2023.120626, 36746576

[31] Gong G Fan J Sun Y Wu Y Liu Y Sun W . Isolation, structural characterization, and antioxidativity of polysaccharide LBLP5-a from *Lycium barbarum* leaves. Process Biochem. (2016) 51:314–24. doi: 10.1016/j.procbio.2015.11.013

[32] Guo W Rao G Wen X. Arabinogalactan in banana: chemical characterization and pharmaceutical effects. Int J Biol Macromol. (2021) 167:1059–65. doi: 10.1016/j.ijbiomac.2020.11.060, 33188809

[33] Liu J Wen XY Kan J Jin CH. Structural characterization of two water-soluble polysaccharides from black soybean (*Glycine max* (L.) Merr.). J Agric Food Chem. (2015) 63:225–34. doi: 10.1021/jf505172m, 25494923

[34] Mou J Yang J Sun Y Liu J Zhao Y Lin H . An arabinogalactan from *Lycium barbarum* mitigated DSS caused intestinal injury via inhibiting mucosal damage and regulating the gut microbiota disorder. Carbohydr Polym. (2025) 352:123155. doi: 10.1016/j.carbpol.2024.123155, 39843060

[35] Rakhmanberdyeva RK Zhauynbayeva KS Senchenkova SN Shashkov AS Bobakulov KM. Structure of arabinogalactan and pectin from the *Silybum marianum*. Carbohydr Res. (2019) 485:107797. doi: 10.1016/j.carres.2019.107797, 31494303

[36] Rodrigues JM Duarte MER Noseda MD. Modified soybean meal polysaccharide with high adhesion capacity to Salmonella. Int J Biol Macromol. (2019) 139:1074–84. doi: 10.1016/j.ijbiomac.2019.08.038, 31398402

[37] Tang S Jiang M Huang C Lai C Fan Y Yong Q. Characterization of arabinogalactans from Larix principis-rupprechtii and their effects on NO production by macrophages. Carbohydr Polym. (2018) 200:408–15. doi: 10.1016/j.carbpol.2018.08.027, 30177181

[38] Wei C Zhang Y He L Cheng J Li J Tao W . Structural characterization and anti-proliferative activities of partially degraded polysaccharides from peach gum. Carbohydr Polym. (2019) 203:193–202. doi: 10.1016/j.carbpol.2018.09.029, 30318204

[39] Wu J Xu Y Zhu B Liu K Wang S Sheng Y . Characterization of an arabinogalactan from the fruit hulls of *Ficus pumila* Linn. And its immunomodulatory effect. J Funct Foods. (2020) 73:104091. doi: 10.1016/j.jff.2020.104091

[40] Xia YG Liang J Yang BY Wang QH Kuang HX. Structural studies of an arabinan from the stems of *Ephedra sinica* by methylation analysis and 1D and 2D NMR spectroscopy. Carbohydr Polym. (2015) 121:449–56. doi: 10.1016/j.carbpol.2014.12.058, 25659720

[41] Yuan L Zhao J Liu Y Zhao J Olnood CG Xu YJ . Multiomics analysis revealed the mechanism of the anti-diabetic effect of Salecan. Carbohydr Polym. (2024) 327:121694. doi: 10.1016/j.carbpol.2023.121694, 38171651

[42] Yuan L Zhong ZC Liu Y. Structural characterisation and immunomodulatory activity of a neutral polysaccharide from Sambucus adnata wall. Int J Biol Macromol. (2020) 154:1400–7. doi: 10.1016/j.ijbiomac.2019.11.021, 31756460

[43] Yuan Y Wang YB Jiang Y Prasad KN Yang J Qu H . Structure identification of a polysaccharide purified from Lycium barbarium fruit. Int J Biol Macromol. (2016) 82:696–701. doi: 10.1016/j.ijbiomac.2015.10.069, 26505952

[44] Zhang H Li C Ding J Lai PFH Xia Y Ai L. Structural features and emulsifying stability of a highly branched arabinogalactan from immature peach (*Prunus persica*) exudates. Food Hydrocoll. (2020) 104:105721. doi: 10.1016/j.foodhyd.2020.105721

[45] Zhang H Zhong J Zhang Q Qing D Yan C. Structural elucidation and bioactivities of a novel arabinogalactan from *Coreopsis tinctoria*. Carbohydr Polym. (2019) 219:219–28. doi: 10.1016/j.carbpol.2019.05.019, 31151520

[46] Zhou L Liao W Chen X Yue H Li S Ding K. An arabinogalactan from fruits of *Lycium barbarum* L. inhibits production and aggregation of Abeta(42). Carbohydr Polym. (2018) 195:643–51. doi: 10.1016/j.carbpol.2018.05.022, 29805023

[47] Liang J Huang YX Zhu XH Zhou FY Wu TY Jia JF . Structural identification, rheological properties and immunological receptor of a complex galacturonoglucan from fruits of Schisandra chinensis (Turcz.) Baill. Carbohydr Polym. (2024) 346:122644. doi: 10.1016/j.carbpol.2024.122644, 39245531

[48] Cao C Liao Y Yu Q Zhang D Huang J Su Y . Structural characterization of a galactoglucomannan with anti-neuroinflammatory activity from Ganoderma lucidum. Carbohydr Polym. (2024) 334:122030. doi: 10.1016/j.carbpol.2024.122030, 38553228

[49] Xu Y Huang C Xu H Xu J Cheng KW Mok HL . Modified Zhenwu decoction improved intestinal barrier function of experimental colitis through activation of sGC-mediated cGMP/PKG signaling. J Ethnopharmacol. (2024) 334:118570. doi: 10.1016/j.jep.2024.118570, 39002824

[50] Zhang W Wang X Yin S Wang Y Li Y Ding Y. Improvement of functional dyspepsia with Suaeda salsa (L.) pall via regulating brain-gut peptide and gut microbiota structure. Eur J Nutr. (2024) 63:1929–44. doi: 10.1007/s00394-024-03401-2, 38703229

[51] Zhai YK Guo XY Ge BF Zhen P Ma XN Zhou J . Icariin stimulates the osteogenic differentiation of rat bone marrow stromal cells via activating the PI3K-AKT-eNOS-NO-cGMP-PKG. Bone. (2014) 66:189–98. doi: 10.1016/j.bone.2014.06.016, 24956021

[52] Li Y Zhang B Liu X Wan H Qin Y Yan H . A bio-inspired nanoparticle coating for vascular healing and immunomodulatory by cGMP-PKG and NF-kappa B signaling pathways. Biomaterials. (2023) 302:122288. doi: 10.1016/j.biomaterials.2023.122288, 37677917

[53] Mohseni AH Casolaro V Bermudez-Humaran LG Keyvani H Taghinezhad SS. Modulation of the PI3K/Akt/mTOR signaling pathway by probiotics as a fruitful target for orchestrating the immune response. Gut Microbes. (2021) 13:1–17. doi: 10.1080/19490976.2021.1886844, 33615993 PMC7899637

[54] Jiang P Ma X Han S Ma L Ai J Wu L . Characterization of the microRNA transcriptomes and proteomics of cochlear tissue-derived small extracellular vesicles from mice of different ages after birth. Cell Mol Life Sci. (2022) 79:154. doi: 10.1007/s00018-022-04164-x, 35218422 PMC11072265

[55] Krachler AM Woolery AR Orth K. Manipulation of kinase signaling by bacterial pathogens. J Cell Biol. (2011) 195:1083–92. doi: 10.1083/jcb.201107132, 22123833 PMC3246894

[56] Sarkar A Hellberg L Bhattacharyya A Behnen M Wang K Lord JM . Infection with *Anaplasma phagocytophilum* activates the phosphatidylinositol 3-kinase/Akt and NF-kappaB survival pathways in neutrophil granulocytes. Infect Immun. (2012) 80:1615–23. doi: 10.1128/IAI.05219-11, 22252875 PMC3318406

[57] Cook SI Sellin JH. Review article: short chain fatty acids in health and disease. Aliment Pharmacol Ther. (1998) 12:499–507. doi: 10.1046/j.1365-2036.1998.00337.x, 9678808

[58] Haskell J. Stress impacts immunity via the gut. Nat Biomed Eng. (2024) 8:1327. doi: 10.1038/s41551-024-01294-4, 39548304

[59] Xue Z Wu C Wei J Xian M Wang T Yang B . An orally administered magnoloside a ameliorates functional dyspepsia by modulating brain-gut peptides and gut microbiota. Life Sci. (2019) 233:116749. doi: 10.1016/j.lfs.2019.116749, 31412264

